# The Role of Ocean Currents in the Temperature Selection of Plankton: Insights from an Individual-Based Model

**DOI:** 10.1371/journal.pone.0167010

**Published:** 2016-12-01

**Authors:** Ferdi L. Hellweger, Erik van Sebille, Benjamin C. Calfee, Jeremy W. Chandler, Erik R. Zinser, Brandon K. Swan, Neil D. Fredrick

**Affiliations:** 1 Department of Civil and Environmental Engineering, Northeastern University, Boston, Massachusetts, United States of America; 2 Grantham Institute & Department of Physics, Imperial College London, London, United Kingdom; 3 ARC Centre of Excellence for Climate System Science, Climate Change Research Centre, University of New South Wales, Sydney, Australia; 4 Department of Microbiology, University of Tennessee, Knoxville, Tennessee, United States of America; 5 Bigelow Laboratory for Ocean Sciences, East Boothbay, Maine, United States of America; University of Connecticut, UNITED STATES

## Abstract

Biogeography studies that correlate the observed distribution of organisms to environmental variables are typically based on local conditions. However, in cases with substantial translocation, like planktonic organisms carried by ocean currents, selection may happen upstream and local environmental factors may not be representative of those that shaped the local population. Here we use an individual-based model of microbes in the global surface ocean to explore this effect for temperature. We simulate up to 25 million individual cells belonging to up to 50 species with different temperature optima. Microbes are moved around the globe based on a hydrodynamic model, and grow and die based on local temperature. We quantify the role of currents using the “advective temperature differential” metric, which is the optimum temperature of the most abundant species from the model with advection minus that from the model without advection. This differential depends on the location and can be up to 4°C. Poleward-flowing currents, like the Gulf Stream, generally experience cooling and the differential is positive. We apply our results to three global datasets. For observations of optimum growth temperature of phytoplankton, accounting for the effect of currents leads to a slightly better agreement with observations, but there is large variability and the improvement is not statistically significant. For observed *Prochlorococcus* ecotype ratios and metagenome nucleotide divergence, accounting for advection improves the correlation significantly, especially in areas with relatively strong poleward or equatorward currents.

## Introduction

Understanding the mechanisms underlying the spatial distribution of organisms is a key part of ecology and evolution [1–3]. Microbes are major players in the global carbon and climate systems, and understanding the present biogeography is a prerequisite for predicting how it may change in the future [4]. For microbes in the surface ocean, both selection by various environmental factors, as well as neutral evolution coupled with dispersal limitation, have been shown to affect the biogeography. However, environmental selection typically exerts a stronger influence [5–8]. There are numerous environmental factors that affect microbe ecology and biogeography, including various nutrients, temperature, light and grazing. Of these, temperature is often invoked to explain ocean microbe biogeography [9–13]. The importance of temperature is also evidenced by its inclusion in models of microbe distribution in the global ocean [14–16].

Here we focus on temperature selection of microbes in the surface ocean. A number of studies have explored the role of temperature in the biogeography of marine microbes by correlating the observed distribution of microbes to temperature [9–13, 17]. One potential limitation with these studies is that they are based on local temperatures. The inherent assumption is that the local temperature is representative of that which led to the selection of the local dominant species or strain. However, ocean currents move water around, which means that the historical conditions experienced by the water at a certain location may be very different from the conditions at that location. For example, microbes in the poleward flowing Gulf Stream off the east coast of the USA experience rapidly decreasing temperatures as they move northward, and the local population may have been shaped by the warmer temperatures of the Gulf of Mexico and Caribbean Sea.

The role of currents and water history have been shown to be important factors in shaping ocean microbe biogeography [18–21]. Early studies of phytoplankton biogeography invoked current transport to explain observations of species at locations with temperatures outside of their native regime [22]. The effect of historical (vs. contemporary) environmental factors has also been explored in the context of light adaption of phytoplankton and found to affect primary productivity in some cases (e.g., turbid coastal waters) [23, 24]. Phytoplankton models that include transport from hydrodynamic models have the effect of currents built in [15, 25]. The role of ocean currents on the temperature record incorporated into the shells of planktic foraminifera has been recognized and quantified using modeling [26, 27]. Over several generations, currents can move microbes across different temperature regions, which increases the range of temperatures they experience by up to 10°C, compared to the seasonal fluctuation at one location [28]. Therefore, the role of currents in ocean microbe biogeography is well-recognized. However, we are not aware of any studies that systematically explored and quantified the effect of ocean currents on temperature selection of microbes and the effect on the observed correlation between microbe distribution and temperature.

We aim to understand and quantify the effect of currents on temperature selection using an individual-based model, where individual microbes with different optimum temperatures are transported based on a hydrodynamic model and compete against each other. We quantify the results using the “advective temperature differential”, the difference between the optimum temperature of the most abundant species of simulations with and without advection. Our results suggest that this differential depends on the location and growth rate and that it correlates with the historical temperature of the water. We apply the results to three global datasets. For observations of optimum growth rates of phytoplankton we find a small but not statistically significant improvement between model and observations when advection is included in the model. For *Prochlorococcus* ecotype ratios and metagenome nucleotide divergence, accounting for currents significantly improves the correlation.

## Methods

### Overview

We model a population of microbes using a general and relatively simple framework that can be applied to phytoplankton and heterotrophic bacteria. Microbe ecology and biogeography is a function of numerous environmental factors (e.g., nutrients, light), but here we are focused on the role of temperature, so other factors are not considered in the model. The model simulates individual cells using an individual-based (a.k.a. agent-based, Lagrangian) approach [29–31]. The individual-based approach can produce substantially different results compared to the more traditional Eulerian approach in many cases. One example is when growth of a heterogeneous population is based on the intracellular nutrient content in a nonlinear manner [30]. Here, growth (division) and death are based on extracellular parameters and for this formulation the individual-based approach produces the same results (i.e., concentration and optimum temperature of the most abundant species) as the Eulerian approach (see SI). However, using individuals allows us to track the temperature history of individuals, which helps to interpret the results. The following summarizes key aspects of the model, with full details provided in S1 File.

A number of global ocean microbe models have been presented [15, 29, 32]. Here, individual cells are transported in the surface layer (top 50m, approximately the depth of the euphotic zone, [33]) on a 2°×2° grid based on advective velocities from a hydrodynamic model [34] and diffusion. Ambient temperatures are taken from the hydrodynamic model, averaged for each 2°×2°×50m grid box at 3-day intervals (see S1 File).

The cells grow (divide) and die, with rates depending on the local population size and temperature (*T*_*loc*_, from the hydrodynamic model). The growth rate is limited by the local population size using a logistic approach, where the carrying capacity is assigned in a spatially and temporally uniform manner, which results in a population with a relatively constant concentration across the globe [29]. This is an implicit way of simulating resource competition, where species “compete for carrying capacity” in place of a resource. We simulate multiple species with different optimum temperatures (see next paragraph for growth rate vs. temperature function).

### Growth rate vs. temperature function

**Fig 1:**
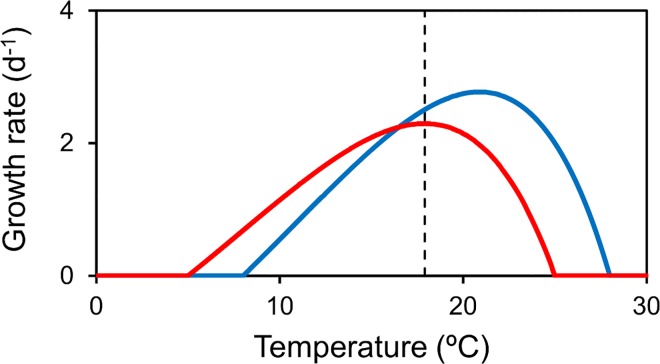
Growth rate vs. temperature. Optimum temperature (*T*_*opt*_) = Red: 18, Blue: 21. See S1 File for parameter values.

For the temperature-dependence of the growth and death rates, we adopt the model of Thomas et al. [9]. The growth rate vs. temperature function, illustrated in Fig 1 for two species, is commonly applied to ocean phytoplankton and well supported by theory and observations [35–37]. The maximum growth rate corresponds to the optimum temperature of the species (*T*_*opt*_, see Table 1 for a summary of the various temperatures used throughout the paper and S1 File for its full formulation). There are a couple of important features of this model. First, the maximum growth rate increases exponentially with optimum temperature (see Eq S7), so that a species with an optimum temperature equal to the local temperature (*T*_*opt*_ = *T*_*loc*_) is actually outcompeted by a species with a slightly larger optimum temperature. For example, compare the growth rates of the red and blue species at the optimum temperature of the red species (dashed line in Fig 1). Second, the curve is asymmetrical with steeper slope above the optimum temperature. That means that, in a variable environment, local temperatures above the optimum are penalized more and it is beneficial to avoid this by having a higher optimum temperature.

**Table 1:** Definition of temperatures

Symbol	Description, notes
*T*_*loc*_	Local temperature
*T*_*opt*_	Optimum temperature, see Fig 1.
*T*_*opt*_*(o)*	Optimum temperature observed for isolated species
*T*_*opt*_*(a)*	Optimum temperature of most abundant species from simulation with advection
*T*_*opt*_*(na)*	Optimum temperature of most abundant species from simulation without advection
*ΔT*_*opt*_	Advective temperature differential, *T*_*opt*_*(a)*–*T*_*opt*_*(na)*
*T*_*hist*_*(g)*	Historical temperature based on growth rate (i.e., lifetime)
*T*_*hist*_*(s)*	Historical temperature based on selection rate
*ΔT*_*hist*_*(s)*	Historical temperature differential based on selection rate, *T*_*hist*_*(s)*–*T*_*loc*_

These features of the growth equation have an important consequence for the effect of temperature on the community composition. Specifically, we generally expect the winning species (i.e., the one with the highest growth rate and abundance) to have an optimum temperature slightly higher than the local temperature, even in the absence of advective transport. This is somewhat counterintuitive, but it is consistent with observations ([9], discussed later in the paper). To quantify the effect of the currents on the optimum temperature, we therefore generally compare the optimum temperature of the most abundant species for simulations with and without advection (i.e., rather than the optimum temperature of the most abundant species vs. local temperature). The difference between these two optimum temperatures is the “advective temperature differential” (*ΔT*_*opt*_ = *T*_*opt*_*(a)*–*T*_*opt*_*(na)*). Turning off or modifying (perturbing) processes in a model to learn about their effect is a relatively common approach. For example, [25] turned off different scales of motion (e.g., mesoscale eddies, vertical mixing) to explore their effect on ocean phytoplankton diversity.

The advective temperature differential is based on the optimum temperature of the most abundant species. For this relatively simple modeling approach, where all species are subjected to the same death rate and transport, abundance is equivalent to time-averaged growth rate and fitness. When there is no advection, the most abundant species (or the optimum temperature of the most abundant species) is a function of the local temperature only. With advection, the most abundant species also reflects the historical temperature experienced by the water upstream. When a sample is collected and characterized in some way (e.g., measure optimum temperature), the results also most likely reflect the most abundant species.

### Historical temperature

To aid in the interpretation of the model results we use the individual-based model to track the temperature history of each individual using an exponential moving average with the growth rate (*T*_*hist*_*(g)*) or selection rate (*T*_*hist*_*(s)*) as weighting parameter. The basic equation for the historical temperature (*T*_*hist*_) is:

$$T_{hist}^{t}=(1-\alpha_{hist}\;\Delta t)T_{hist}^{t-\Delta t}+\alpha_{hist}\;\Delta t\;T_{loc}^{t}$$ (1)

*α*_*hist*_ (d^-1^) is the weighting parameter that characterizes the length of memory. Larger values will give more weight to the current temperature (less memory) and smaller values will give more weight to the historical temperature (more memory). For the weighting parameter, we use the growth or selection rates, both of which change with time and space, as described below. The integration time step (*Δt* = 0.3 d) is chosen so that the term *α*_*hist*_ *Δt* is between 0 and 1.

One approach is to use the growth rate (*k*_*g*_, d^-1^) as weighting parameter. Then, *T*_*hist*_*(g)* is an estimate of the temperature experienced during the lifetime of the cell. More specifically, it is the historical temperature weighted by the biomass synthesized at that temperature. Consider, for example, a cell that grows at *T*_*loc*_ = 15°C for a long time and then at *T*_*loc*_ = 20°C at *k*_*g*_ = 0.5/d for 3 d. The fraction of biomass synthesized at *T*_*loc*_ = 15°C and 20°C is 0.22 (exp[-*k*_*g*_ *t*]) and 0.78 (1–0.22), respectively. The weighted average temperature is *T*_*hist*_*(g)* = 19°C. The same result is obtained by integrating the above equation over multiple time steps (*Δt* = 0.3 d).

Another approach is to use the selection rate (*s*, d^-1^) as weighting parameter. The selection rate is defined as the difference between the growth rates of species in competition, and it is most often applied to two species. Here, we have many species and we define the selection rate as the difference between the growth rate of the optimum species (i.e., the one with the highest concentration) and that of the adjacent species (i.e., the one with the second-highest concentration). Calculated in this manner, the selection rate characterizes how fast the dominant species is changing. For this study, where we are generally concerned with the optimum temperature of the most abundant species, this is an appropriate measure of the memory time-scale of the population.

### Simulations performed

Typical simulations are run for 31 years with 50 species (with *T*_*opt*_ from -5.2 to 36.2°C in 0.8°C steps, which covers the range of ambient temperatures) for a total of 25 million cells and growth rate parameter adjusted to obtain global population average growth rates as desired (e.g., 0.14 d^-1^ for heterotrophic bacteria, [38], [39]). See the S1 File for additional discussion on parameter values.

## Results and Discussion

### Effect of currents on temperature selection

**Fig 2:**
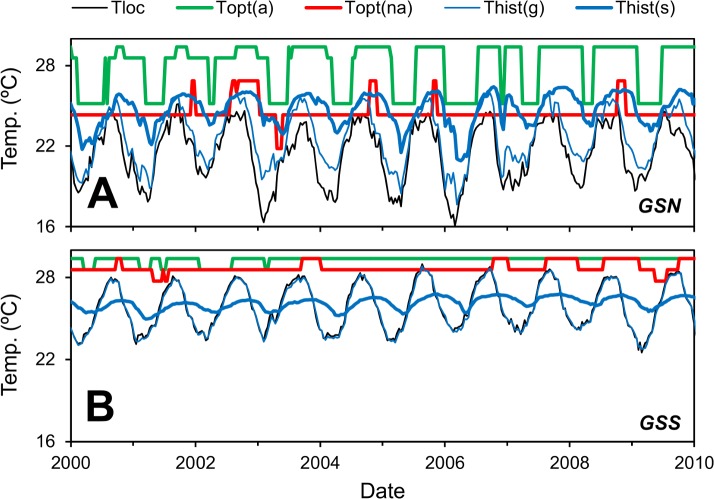
Time series of model results. Local temperature (*T*_*loc*_), optimum temperatures of the most abundant species in simulations with (*T*_*opt*_*(a)*) and without(*T*_*opt*_*(na)*) advection and historical temperature based on growth (*T*_*hist*_*(g)*) and selection (*T*_*hist*_*(s)*) rates at two locations in the Gulf Stream (GSS and GSN in Fig 3). Population average growth rate = 0.14 d^-1^.

**Fig 3:**
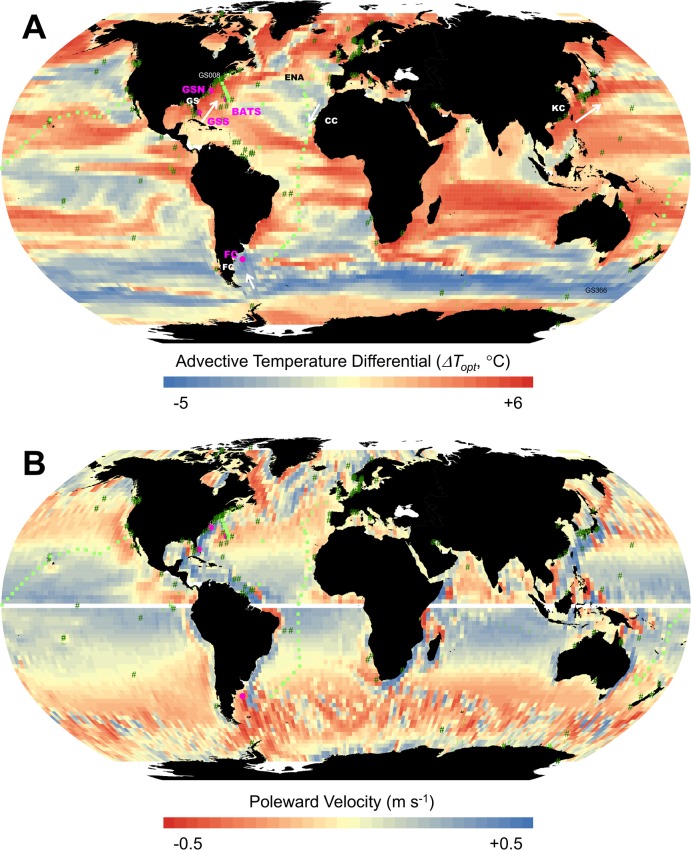
Map of model results. (A) Advective temperature differential (*ΔT*_*opt*_) across the global ocean, defined as the difference between optimum temperatures of the most abundant species in simulation with and without advective transport. Population average growth rate = 0.14 d^-1^. Values are averages over the 31-year simulation period. Also shown are locations used in Figs 2 and 4 (pink circles and text), isolation locations for phytoplankton in Fig 5 (dark green triangles), *Prochlorococcus* ecotypes in Fig 6 (light green squares, open symbols are for Gulf Stream (GS) and eastern North Atlantic (ENA) samples, also identified in Fig 6.) and metagenome nucleotide divergence in Fig 7 (medium green circles, samples GS008 and GS366 are labelled). Approx. location of select currents (white arrows and white bold text). GS = Gulf Stream, FC = Falkland Current, CC = Canary Current, KC = Kuroshio Current. (B) Poleward velocity. See S1 File for a larger version of the North Atlantic.

Before taking the global view of the effect of advection on temperature selection, we present temperatures for simulations with and without advection for two locations in the Gulf Stream (Fig 2, see Fig 3 for locations). The model output is presented as the optimum temperature of the most abundant species. The local and optimum temperatures of the most abundant species exhibit similar patterns, but there are a number of differences. First, the optimum temperature of the most abundant species changes less smoothly. At the northern location (GSN), at least two species with temperature optima of about 25 and 29°C co-exist. Note that the difference in temperature optima of adjacent species in the model is less than 1°C, so this relatively large difference is not a result of a low number of species included in the model, but reflects the selection dynamics of the system. The species with the highest instantaneous growth rate changes frequently and smoothly follows the local temperature. However, for most species this only lasts for a few weeks, which is not long enough to rise to dominance (see S1 File for further discussion). Second, as discussed above, the optimum temperature of the most abundant species is generally higher than the local temperature, even for the case of no advection. Third, the optimum temperature of the most abundant species lags the local temperature, because it takes some time for the species with the new optimum temperature to outcompete the one with the old optimum temperature. The local temperature is higher at the southern, upstream location (GSS). Consequently, the population at the northern, downstream location (GSN) has experienced a warmer temperature and that affects the optimum temperature.

**Fig 4:**
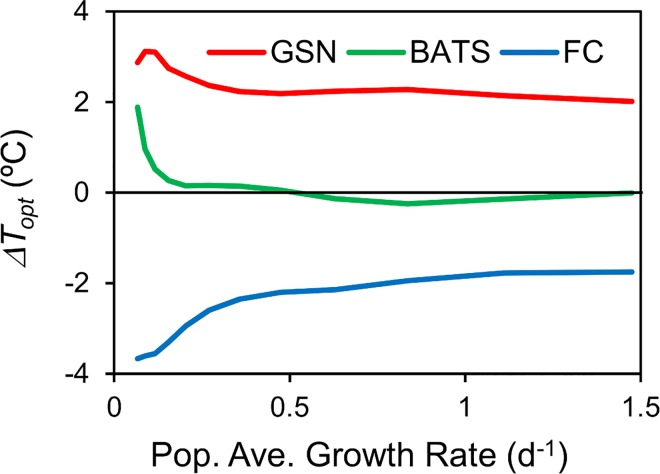
Direction and magnitude of advective temperature differential depends on location and growth rate. Difference of optimum temperature of the most abundant species in simulations with and without advection (*ΔT*_*opt*_) for locations in the Gulf Stream (GSN in Fig 3), Bermuda Atlantic Time Series (BATS in Fig 3) and Falkland Current (FC in Fig 3) for different growth rates. Values are averages over the 31-year simulation period.

The advective temperature differential (*ΔT*_*opt*_) for the GSN site is about 3.2°C (see also Fig 4 discussed subsequently), meaning that incorporating advection by ocean currents leads to over 3°C higher optimum temperature of the most abundant species. At first glance it may be a bit surprising that there is such a large difference for a relatively fast-growing microbe population. The generation time of heterotrophic bacteria is about 5 days and the historical temperature based on growth rate at the GSN location is close to the local temperature (see *T*_*loc*_ and *T*_*hist*_*(g)* in Fig 2A). The historical temperature based on growth rate is an estimate of the temperature experienced by a cell over its lifetime (see Methods) and is appropriate to use when one is concerned with the temperature record of individuals, as in shells of planktic foraminifera [26]. However, the optimum temperature of the most abundant species at any location is a property of the microbe community, rather than the individual microbes. When the temperature changes, it may take several generations before this translates into a change in community composition. A more appropriate measure to characterize the time scale of temperature memory is the selection rate, the difference in growth rates between competing species. The historical temperature calculated in this manner (*T*_*hist*_*(s)*) is substantially above the local temperature at this location. In fact, the historical temperature differential (*ΔT*_*hist*_*(s)*, see Table 1) is about 3.1°C, which is close to the advective temperature differential (*ΔT*_*opt*_). At the global scale, the historical temperature differential parameter can explain much of the model results in advective temperature differential (see S1 File).

The direction (warmer or colder) and magnitude of the advective temperature differential (defined as the difference in optimum temperature of the most abundant species of simulations with and without advection) is a function of the location and growth rate (Fig 4). Locations with strong poleward currents have populations that experience a cooling and therefore have a positive differential (Gulf Stream, Figs 2 and 4), whereas locations with strong equatorward currents have a negative differential (Falkland Current, Fig 4). The magnitude of the differential depends on the growth rate. For microbes with relatively low growth rates (~0.14 d^-1^, characteristic of heterotrophic bacteria, [38], [39]), the selection rate is lower and the population has a longer memory and larger differential (Fig 4). Populations with higher growth rates (~0.65 d^-1^, characteristic of phytoplankton, [38], [39]) have shorter memory and smaller differential (Fig 4). Simulations with even higher growth rates show the differentials continuing to converge towards zero.

A global map of advective temperature differential (*ΔT*_*opt*_) illustrates that this differential can be quite heterogeneous across the globe (Fig 3A). As for the individual locations (Fig 4), the direction and magnitude depends on the location. Poleward-flowing western boundary currents, like the Gulf Stream and Kuroshio Current, have positive differential. Equatorward-flowing currents, like the Falkland Current and Canary Current, have negative differential. These global patterns are also qualitatively similar to those for temperature offsets for drift of planktic foraminifera presented by van Sebille et al. [26] and the inter-generational temperature ranges due to drift experienced by microbes presented by Doblin and van Sebille [28]. However, these two previous studies did not explicitly consider fitness and selection. A map of poleward velocities shows a pattern that is consistent with this (Fig 3B).

Fig 3 presents results for a growth rate characteristic of heterotrophic bacteria (~0.14 d^-1^, [38], [39]). We present maps of all temperature variables (see Table 1) for a range of growth rates (0.07–1.3 d^-1^), an atlas of selection temperatures, in S2 File. Those growth rates cover the range typical of heterotrophic bacteria (0.14 d^-1^) and phytoplankton (0.65 d^-1^) in the ocean [38, 39]. Note, however, that for some species growth rates can be even lower, and may approach zero if we consider dormancy [40]. Also, zooplankton may have lower growth rates. For example, in their model of planktic foraminifera [26] used lifespans of up to 180 days, corresponding to growth rates of 0.004 d^-1^.

### Comparison to observations: Phytoplankton optimum temperatures

Our results suggest that the effect of advection can be substantial in currents that experience strong warming or cooling, which means that explicitly considering how currents carry planktonic organisms should improve observed correlations between microbe distributions and temperature. We explore this using experimentally-determined optimum temperatures for phytoplankton from two datasets. The dataset of Thomas et al. [9] includes observations from 194 marine and estuarine isolates. Here, we included the 153 observations identified as marine. The dataset of Chen et al. [10] includes 220 observations of marine phytoplankton. Note that both datasets are based on a literature review and they are not mutually exclusive (i.e., several observations are included in both datasets). However, they were developed using different regression methods for estimating the optimum temperature. The locations are shown in Fig 3. For the model simulations, the growth rate parameter was adjusted so that the global average growth rate was about 0.65 d^-1^, consistent with estimates for phytoplankton [38].

**Fig 5:**
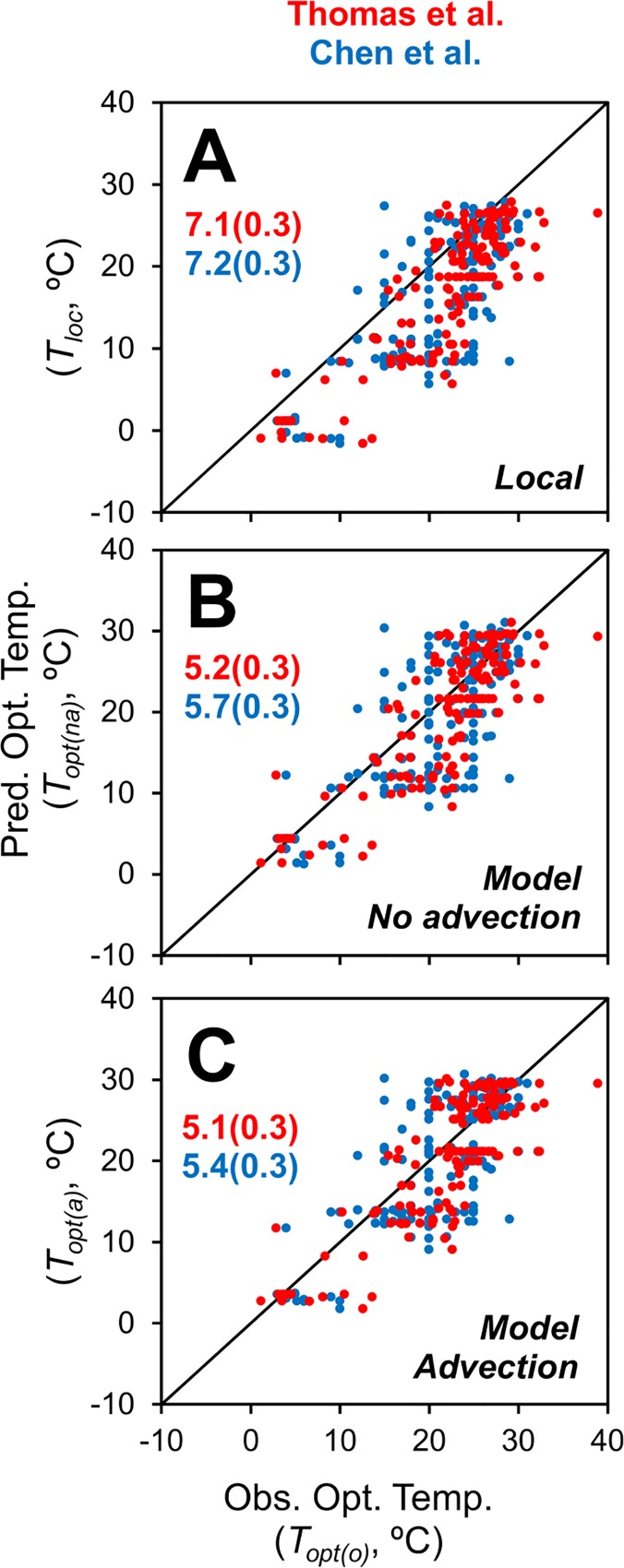
Model–data comparison for phytoplankton optimum temperatures. Predicted vs. observed optimum temperatures for the datasets of Thomas et al. [9] and Chen et al. [10]. (A) Prediction is local temperature. (B) Prediction from model without advection. (C) Prediction from model with advection. Numbers on panels are RMSEs (±SD, bootstrap analysis, *n* = 1,000).

The observed optimum temperature (*T*_*opt*_*(o)*) is generally higher than the local temperature (*T*_*loc*_, from the hydrodynamic model) (Fig 5A). This can be explained by the changes in maximum growth rate and asymmetry of the growth rate vs. temperature relationship (Fig 1), as discussed above.

The comparison of observed optimum temperature (*T*_*opt*_*(o)*) to optimum temperatures of the most abundant species in the model without advection (*T*_*opt*_*(na)*) is better (Fig 5B). The difference between the root-mean-square error (RMSE) values in panels A and B of Fig 5 is statistically significant (bootstrap analysis, based on difference of RMSE values, *n* = 1,000, *p* < 0.001). The temperature function used in the model predicts a higher optimum temperature, consistent with the observations. This model is similar to that of Thomas et al. [9] and the results are consistent.

The comparison to optimum temperatures of the most abundant species in the model with advection (*T*_*opt*_*(a)*) is similar. For both datasets, the model with advection has a slightly lower RMSE. However, the differences between these RMSE values are low and not statistically significant (p < 0.05). There are a couple of possible reasons for this. First, there is substantial variability in the observations. For example, in the Thomas et al. dataset, one location near the southern end of Japan has 26 samples with observed optimum temperatures ranging from 21 to 32°C. In the Chen et al. dataset, the three samples at BATS have observed optimum temperatures ranging from 18 to 30°C. This local co-existence of species with different optimum temperatures is also predicted by the model, which suggests it is due to seasonal dynamics (see Fig 2 and associated discussion). Therefore, the variability in the observations is not due to an error *per se*, but it nonetheless overshadows the relatively small difference between the models with and without advection. Second, the Thomas et al. and Chen et al. datasets include predominantly coastal isolates (i.e., vs. open-ocean, see Fig 3). The dynamics of the circulation in these coastal regions may be substantially different from the open ocean, and may not be completely resolved by the 1/10° resolution global hydrodynamic model. We explored using various subsets of the database, including lower local temperatures (*T*_*loc*_ < 20°C) and various functional groups (e.g., diatoms, cyanobacteria, based on Thomas et al. [41]), but this did not result in a statistically significant difference between these two models.

### Comparison to observations: *Prochlorococcus* ecotype ratios

**Fig 6:**
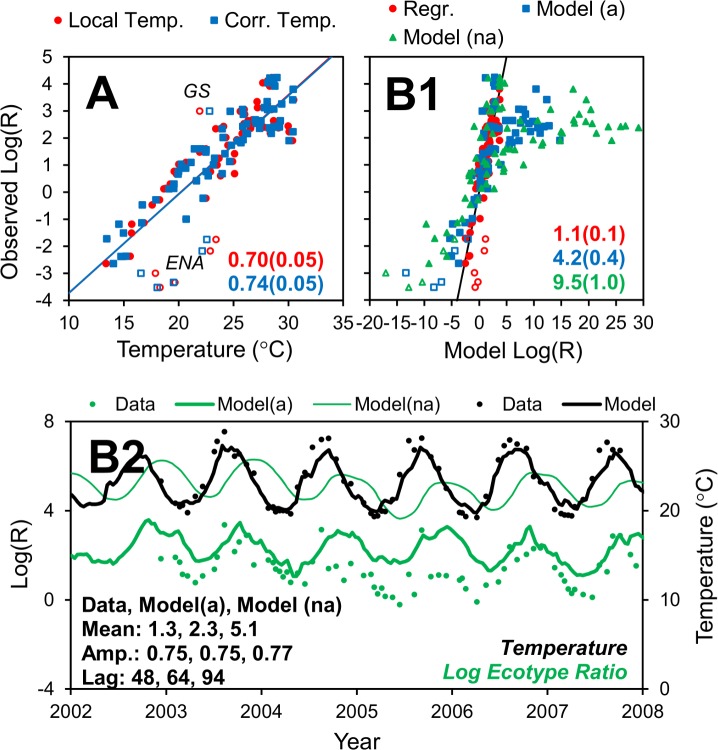
Model–data comparison for *Prochlorococcus* ecotypes. (A) Regression of observed log ecotype ratio to local and corrected temperatures. The corrected temperature was calculated as the local temperature (*T*_*loc*_) plus the advective temperature differential (*ΔT*_*opt*_) from the atlas (see SI). Open symbols are for Gulf Stream (GS) and eastern North Atlantic (ENA) samples, also identified in Fig 3. (B) Direct prediction of log ecotype ratio using ecotype-specific growth rate vs. temperature functions (see SI). (B1) Observed vs. modeled log ecotype ratio. (B2) Observed and modeled log ecotype ratio vs. time at BATS. Numbers on panel A are R^2^s (±SD, bootstrap analysis, *n* = 1,000) and B1 are RMSEs (±SD, bootstrap analysis, *n* = 1,000). Numbers on panel B2 are parameters of sine curve fit to log ecotype ratios in observed (Data), model with advection (Model(a)) and model without advection (Model(na)). Lag is relative to temperature.

We also apply our results to the dataset of *Prochlorococcus* ecotype ratios from Chandler et al. [42]. This dataset covers regions in the Atlantic and Pacific Ocean, including transects that spanned both northern and southern hemispheres of both oceans. *Prochlorococcus* is a genus of marine cyanobacteria that dominates the picophytoplankton community of the oligotrophic oceans. There are numerous strains or ecotypes that are adapted to different temperature, light and nutrient conditions, which control their biogeography [5, 8, 15, 43–46]. The high-light adapted ecotypes eMIT9312 and eMED4 numerically dominate the surface layer with the former dominating the warmer, lower latitudes, and the latter dominating the cooler, higher latitudes [8, 42]. Interestingly, these two ecotypes do not compete to the exclusion of the other, and their co-existence can be described as a log-linear increase in eMIT9312 relative to eMED4 as a function of increasing temperature ([42], Fig 6A, red symbols). The largest discrepancies between the observations and the regression are for one sample in the Gulf Stream and five samples in the eastern North Atlantic (labelled GS and ENA in Fig 6A, see Fig 3 for locations). At these locations, there are relatively strong poleward (GS) and equatorward currents (ENA), which suggests that accounting for advection can improve the correlation. We use the results from our model to “correct” the temperatures using the advective temperature differential provided in the atlas. Using the corrected temperatures improves the correlation with the observed log ecotype ratio (Log(R)) significantly (Fig 6A, blue symbols, bootstrap analysis, based on difference of R^2^ values, *n* = 1,000, *p* < 0.001). The GS and ENA data points make up five out of the six largest improvements in the regression. Although the improvement is evident and consistent with expectations, the correction does not go so far as to bring these data points in line with the rest of the population.

Another approach is to directly simulate the *Prochlorococcus* ecotypes using growth rate vs. temperature functions based on data from laboratory experiments with the eMIT9312 and eMED4 ecotypes (see SI). For this analysis the transect data are presented as observed vs. modeled log ecotype ratio (Fig 6B1). As a baseline, the regression equation in panel A (uncorrected) is used to predict ecotype ratios. This is just another way of looking at the same information already presented in panel A. There is a good agreement between the observations and the predictions using the correlation. The direct simulation does relatively well considering it is a straight (i.e., uncalibrated) prediction. However, it does worse than the regression model. This suggests that there are other environmental factors that correlate with temperature (e.g., light, nutrients, grazer activity) that also affect the ecotype ratio. The effect of those factors are implicitly captured by the empirical regression model, but not the direct simulation, which uses growth rate vs. temperature functions based on observations from laboratory experiments that exclude other factors. This is an area of future research and can be explored by including additional factors in this model or modifying existing ecosystem models. For example, the model of Follows et al. [15] can already explain much of the global biogeography of eMIT9312 and eMED4 using a number of nutrients (phosphorus, nitrogen, iron and silica), light and temperature. That model accounts for the history of environmental variables, but simulates the effect of temperature using a more general equation. It could be modified to include the more specific growth rate vs. temperature functions used here and applied to this dataset (i.e., Fig 6), which would allow for the effect of the various environmental factors to be quantified (e.g., by turning factors on/off). The purpose of the present project is to explore the effect of advection. If advection is turned off in the model, the agreement between the model and observations decreases significantly (bootstrap analysis, based on difference of RMSE values, *n* = 1,000, *p* < 0.001).

We also compare the model to observations from the BATS time series (Fig 6B2). The observations show a time lag between the local temperature and the log ecotype ratio of about 48 days (obtained by fitting a sine curve to the temperature and ecotype time series). The model ecotype ratio is generally higher, has a lower amplitude and longer time lag (with temperature), again suggesting that there are other factors that contribute to the observed ecotype ratio. When the advection is turned off, the agreement with the model and observations decreases substantially.

### Comparison to observations: Metagenome nucleotide divergence

**Fig 7:**
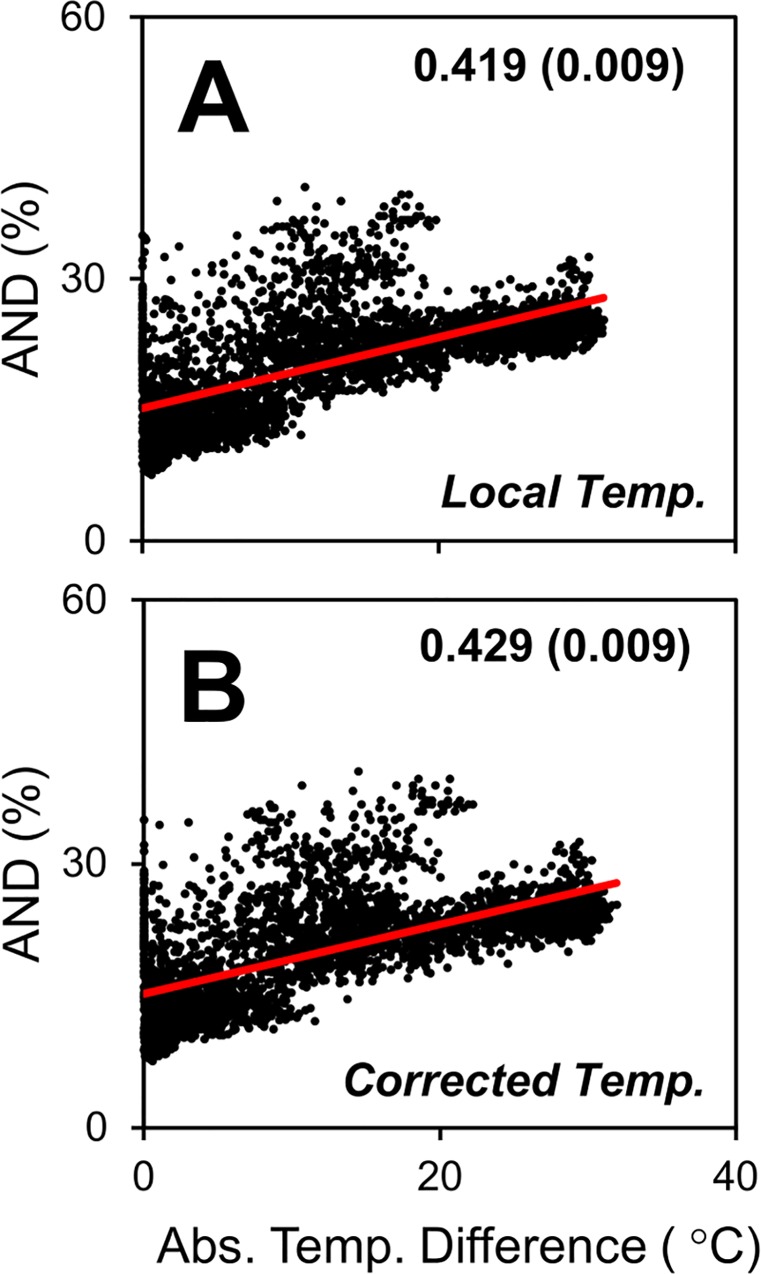
Model–data comparison for metagenome nucleotide divergence. Regression of average nucleotide divergence (AND) to absolute temperature difference of samples based on (A) local and (B) corrected temperatures. The corrected temperature was calculated as the local temperature (*T*_*loc*_) plus the advective temperature differential (*ΔT*_*opt*_) from the atlas (see SI). Population average growth rate = 0.14 d^-1^. Numbers on panels are R^2^s (±SD, bootstrap analysis, *n* = 1,000).

Finally, we apply our results to a surface ocean metagenome dataset [13]. This dataset includes 87 samples from the surface ocean for which the DNA was sequenced. There are numerous bioinformatics methods available to analyze these raw DNA sequences, such as whole genome alignment or recruitment (akin to *in silico* DNA hybridization). Several past studies have explored the role of temperature in marine metagenomics datasets [11–13, 17] and found it to be an important variable, making this dataset an ideal test case here. In this dataset, the 87 metagenome samples are compared against each other (87 × 87 = 7,569 data points). Their difference is quantified as average nucleotide divergence (AND). Metagenome sequences were quality processed using PRINSEQ [47], and all sequences with the following characteristics were removed from further analysis: sequences <100 bp, sequences containing any ambiguities (Ns), all forms of replicate and duplicate sequences, and sequences with a minimum entropy value of 70 (applied to pyrosequencing datasets only). The bioinformatic tool Mash [48] was used to estimate pairwise AND values of each metagenome pair. Then, AND was correlated to the absolute temperature difference of the sample pair (Fig 7). The slope is positive, meaning sample pairs with larger difference in temperature have larger AND, which is intuitively correct. The temperature difference calculated using the local temperatures can explain about 42% of the variance in AND. When the temperature difference is calculated using the corrected temperatures, it can explain 43%. This is a small, but significant improvement (bootstrap analysis, based on difference of R^2^ values, *n* = 1,000, *p* < 0.001). The largest improvement is seen for the sample pair GS008 (Newport Harbor, USA) and GS366 (Southern Ocean), which are from areas of positive and negative advective temperature differential, respectively (see Fig 3A).

## Summary and Outlook

We explored the role of advection on temperature selection of microbes in the global surface ocean using an individual-based model. Our results suggest that currents can distort temperature selection: In areas with substantial warming or cooling currents, the local temperature is not a good indicator of the historical temperature experienced by the microbial community. The direction and magnitude of the advective temperature differential depends on the location and growth rate of the microbes. In poleward currents like the Gulf Stream, the addition of advection by ocean currents leads to a relatively high increase of the optimum temperature of the most abundant species. For microbes with faster growth and selection rates the memory is shorter and the differential lower, compared to those with slower growth rates.

Comparison of the model to observed optimum temperature for phytoplankton suggests that accounting for currents results in a small improvement, but the difference is not statistically significant. This is likely because the variability in the observations is very high to begin with, so that the signal-to-noise level in our analysis is low. More observations, collected using standard protocols (e.g. [49]) and also farther into the open ocean, may help resolve some of these discrepancies. Our model results could help select locations where we expect a substantial effect, including in the Southern Ocean and the extensions of the Western Boundary Currents (Fig 3).

Comparison to observations of *Prochlorococcus* ecotype ratios shows that accounting for advection improves the correlation. Future improvements in understanding the biogeography of *Prochlorococcus* will likely require accounting for additional variables, like light and nutrients, which can have ecotype-specific effects on growth and physiology [45, 50–53]. Importantly, the impacts of light level and nutrient concentration on the growth rates of *Prochlorococcus* as a function of temperature have yet to be investigated in laboratory cultures. Of course, advection will also play a role for those variables, which can be accounted for in the same manner as we did for temperature here.

The observed nucleotide divergence of metagenome sample pairs correlates positively with their absolute temperature difference. Basing the temperature difference on temperatures corrected for advection improves the correlation.

In summary, the applications to phytoplankton temperature optima, *Prochlorococcus* ecotypes and metagenome nucleotide divergence datasets suggest that temperature biogeography studies would benefit if they account for advection. In terms of R^2^, or percent of variance explained, the correlation to temperature improves on average about 3% if advection is considered (Table 2). We hope that future studies will utilize and benefit from the atlas of temperature corrections we developed and present in S2 File.

**Table 2:** Improvement when including advection

Dataset	ΔR^2^ (%) (a)
Phytoplankton optimum temperatures	+1.46
(Thomas et al.)	(+0.24)
(Chen et al.)	(+2.68)
*Prochlorococcus* ecotypes	+6.17
Metagenome nucleotide divergence	+2.23
***Average***	**+3.29**

(a) Relative improvement of correlation to temperature. Calculated as [(R^2^ considering advection)–(R^2^ not considering advection)] / (R^2^ not considering advection) × 100.

There are a number of potential improvements and follow-on studies. Our model and analysis is limited to 2D surface currents and temperature. However, vertical advection (e.g., upwelling) will also likely affect temperature selection and this could be explored using a 3D analysis. We use a spatially and temporally constant carrying capacity. Gradients in population size (e.g., coastal vs. open ocean, across fronts) may change the influences of upstream vs. local populations (mass effects, [54]). Blooms, eddies and mixing events may also affect temperature selection at times. These effects are expected to be less when averaged across time and along major currents in the open ocean, but it would be useful to explore this further using a model with variable carrying capacity. It would be straightforward to base the carrying capacity on chlorophyll maps from satellites. Modeling variable population sizes is relatively simple using the Eulerian approach, but more difficult when simulating individuals [55]. Also, currents will not only affect temperature, but other variables, like various nutrients and light levels. Understanding the role of currents on the effect of these variables will require the use of a biogeochemical model that explicitly resolves them [15, 25]. Our model explores the selection of microbes, but does not consider adaptation or evolution. The time scales of these processes are longer than the immediate response of the growth rate on the local temperature included in our model, but they may be relevant considering travel times in ocean currents. How important adaptation and evolution are in the biogeography of marine microbes is an open question. The individual-based approach is well-suited for and could be used to simulate these processes [16, 56].

Finally, the growth and death formulations in our model are relatively simple (i.e., basic functions of temperature and local population size). However, the model can be used as a starting point for the development of more mechanistically-detailed models. Much more is known about microbial biology and ecology, and biogeochemistry and biogeography studies need to take advantage of this knowledge. We are presently combining this model with an existing gene-level model of *Synechococcus* [57] and plan to compare model predictions to observations of transcript levels [58].

## Supporting Information

S1 File Supporting Text, Figures and Tables. This file includes Model description, Selection dynamics, Plankton datasets notes, Additional model results and References.
(PDF)

S2 File Atlas. The atlas is presented as maps in PDF format (ATLASMAPS) and an MS Excel book (ATLASTABLE) and accompanying ESRI shapefile (ATLASGIS). The atlas includes results from simulations with different average growth rates. Columns/attributes are named using the variables used throughout the paper. The average growth rate is appended to the column name. For example, TOPTA028 is *T*_*opt*_*(a)* from the simulation with average growth rate of 0.28 d^-1^.
(ZIP)

S1 Movie Movie. The movie has two panels. The left panel shows the local temperature. The right panel shows individual cells colored by their optimum temperature. Simulation with advection. In this simulation the number of individuals was reduced to ~50,000 to allow for visualization of individuals.
(AVI)

## References

[1] Hanson CA, Fuhrman JA, Horner-Devine MC, Martiny JBH. Beyond biogeographic patterns: processes shaping the microbial landscape. Nat Rev Micro. 2012;10(7):497–506. 10.1038/nrmicro2795 22580365

[2] Brown MV, Ostrowski M, Grzymski JJ, Lauro FM. A trait based perspective on the biogeography of common and abundant marine bacterioplankton clades. Marine Genomics. 2014;15:17–28. 10.1016/j.margen.2014.03.002 24662471

[3] Ramette A, Tiedje J. Biogeography: An Emerging Cornerstone for Understanding Prokaryotic Diversity, Ecology, and Evolution. Microb Ecol. 2007;53(2):197–207. 10.1007/s00248-005-5010-2 17106806

[4] Falkowski PG, Barber RT, Smetacek V. Biogeochemical Controls and Feedbacks on Ocean Primary Production. Science. 1998;281(5374):200–6. 9660741 10.1126/science.281.5374.200

[5] Martiny AC, Tai APK, Veneziano D, Primeau F, Chisholm SW. Taxonomic resolution, ecotypes and the biogeography of Prochlorococcus. Environmental Microbiology. 2009;11(4):823–32. 10.1111/j.1462-2920.2008.01803.x 19021692

[6] Pommier T, Douzery EJP, Mouillot D. Environment drives high phylogenetic turnover among oceanic bacterial communities. Biology Letters. 2012;8(4):562–6. 10.1098/rsbl.2011.0990 22258446 PMC3391433

[7] Sul WJ, Oliver TA, Ducklow HW, Amaral-Zettler LA, Sogin ML. Marine bacteria exhibit a bipolar distribution. Proceedings of the National Academy of Sciences. 2013;110(6):2342–7. 10.1073/pnas.1212424110 PMC3568360 23324742

[8] Johnson ZI, Zinser ER, Coe A, McNulty NP, Woodward EMS, Chisholm SW. Niche Partitioning Among Prochlorococcus Ecotypes Along Ocean-Scale Environmental Gradients. Science. 2006;311(5768):1737–40. 10.1126/science.1118052 16556835

[9] Thomas MK, Kremer CT, Klausmeier CA, Litchman E. A Global Pattern of Thermal Adaptation in Marine Phytoplankton. Science. 2012;338(6110):1085–8. 10.1126/science.1224836 23112294

[10] Chen B, Liu H, Huang B, Wang J. Temperature effects on the growth rate of marine picoplankton. Marine Ecology Progress Series. 2014;505:37–47.

[11] Dupont CL, Rusch DB, Yooseph S, Lombardo M-J, Alexander Richter R, Valas R, et al Genomic insights to SAR86, an abundant and uncultivated marine bacterial lineage. ISME J. 2012;6(6):1186–99. 10.1038/ismej.2011.189 22170421 PMC3358033

[12] Brown MV, Lauro FM, DeMaere MZ, Muir L, Wilkins D, Thomas T, et al Global biogeography of SAR11 marine bacteria. Molecular Systems Biology. 2012;8(1):n/a-n/a. 10.1038/msb.2012.28 PMC3421443 22806143

[13] Swan BK, Tupper B, Sczyrba A, Lauro FM, Martinez-Garcia M, González JM, et al Prevalent genome streamlining and latitudinal divergence of planktonic bacteria in the surface ocean. Proceedings of the National Academy of Sciences. 2013. 10.1073/pnas.1304246110 PMC3710821 23801761

[14] Flombaum P, Gallegos JL, Gordillo RA, Rincón J, Zabala LL, Jiao N, et al Present and future global distributions of the marine Cyanobacteria Prochlorococcus and Synechococcus. Proceedings of the National Academy of Sciences. 2013;110(24):9824–9. 10.1073/pnas.1307701110 PMC3683724 23703908

[15] Follows MJ, Dutkiewicz S, Grant S, Chisholm SW. Emergent Biogeography of Microbial Communities in a Model Ocean. Science. 2007;315(5820):1843–6. 10.1126/science.1138544 17395828

[16] Toseland A, Daines SJ, Clark JR, Kirkham A, Strauss J, Uhlig C, et al The impact of temperature on marine phytoplankton resource allocation and metabolism. Nature Clim Change. 2013;3(11):979–84. http://www.nature.com/nclimate/journal/v3/n11/abs/nclimate1989.html#supplementary-information.

[17] Gianoulis TA, Raes J, Patel PV, Bjornson R, Korbel JO, Letunic I, et al Quantifying environmental adaptation of metabolic pathways in metagenomics. Proceedings of the National Academy of Sciences. 2009;106(5):1374–9. 10.1073/pnas.0808022106 PMC2629784 19164758

[18] Galand PE, Potvin M, Casamayor EO, Lovejoy C. Hydrography shapes bacterial biogeography of the deep Arctic Ocean. ISME J. 2010;4(4):564–76. 10.1038/ismej.2009.134 20010630

[19] Hamdan LJ, Coffin RB, Sikaroodi M, Greinert J, Treude T, Gillevet PM. Ocean currents shape the microbiome of Arctic marine sediments. ISME J. 2013;7(4):685–96. 10.1038/ismej.2012.143 23190727 PMC3603395

[20] Agogue H, Lamy D, Neal PR, Sogin ML, Herndl GJ. Water mass-specificity of bacterial communities in the North Atlantic revealed by massively parallel sequencing. Molecular Ecology. 2011;20(2):258–74. 10.1111/j.1365-294X.2010.04932.x 21143328 PMC3057482

[21] Fuhrman JA, Steele JA. Community structure of marine bacterioplankton: patterns, networks, and relationships to function. Aquatic Microbial Ecology. 2008;53(1):69.

[22] Smayda TJ. Biogeographical Studies of Marine Phytoplankton. Oikos. 1958;9(2):158–91.

[23] Falkowski PG, Wirick CD. A simulation model of the effects of vertical mixing on primary productivity. Marine Biology. 1981;65(1):69–75.

[24] Nagai T, Yamazaki H, Kamykowski D. A Lagrangian photoresponse model coupled with 2nd-order turbulence closure. Marine Ecology Progress Series. 2003;265:17–30.

[25] Lévy M, Jahn O, Dutkiewicz S, Follows MJ. Phytoplankton diversity and community structure affected by oceanic dispersal and mesoscale turbulence. Limnology and Oceanography: Fluids and Environments. 2014;4(1):67–84.

[26] van Sebille E, Scussolini P, Durgadoo JV, Peeters FJC, Biastoch A, Weijer W, et al Ocean currents generate large footprints in marine palaeoclimate proxies. Nat Commun. 2015;6. 10.1038/ncomms7521 25735516

[27] Weyl PK. Micropaleontology and Ocean Surface Climate. Science. 1978;202(4367):475–81. 10.1126/science.202.4367.475 17813469

[28] Doblin MA, van Sebille E. Drift in ocean currents impacts intergenerational microbial exposure to temperature. Proceedings of the National Academy of Sciences. 2016;113(20):5700–5. 10.1073/pnas.1521093113 PMC4878470 27140608

[29] Hellweger FL, van Sebille E, Fredrick ND. Biogeographic patterns in ocean microbes emerge in a neutral agent-based model. Science. 2014;345(6202):1346–9. 10.1126/science.1254421 25214628

[30] Bucci V, Nunez-Milland D, Twining B, Hellweger F. Microscale patchiness leads to large and important intraspecific internal nutrient heterogeneity in phytoplankton. Aquat Ecol. 2012;46(1):101–18.

[31] Woods JD. The Lagrangian Ensemble metamodel for simulating plankton ecosystems. Progress in Oceanography. 2005;67(1–2):84–159.

[32] Clark JR, Lenton TM, Williams HT, Daines SJ. Environmental selection and resource allocation determine spatial patterns in picophytoplankton cell size. Limnol Oceanogr. 2013;58(3):1008–22.

[33] Lee Z, Weidemann A, Kindle J, Arnone R, Carder KL, Davis C. Euphotic zone depth: Its derivation and implication to ocean-color remote sensing. Journal of Geophysical Research: Oceans. 2007;112(C3):n/a-n/a.

[34] Masumoto Y, Sasaki H, Kagimoto T, Komori N, Ishida A, Sasai Y, et al A fifty-year eddy-resolving simulation of the world ocean: Preliminary outcomes of OFES (OGCM for the Earth Simulator). J Earth Simulator. 2004;1:35–56.

[35] Kingsolver J. The Well‐Temperatured Biologist. The American Naturalist. 2009;174(6):755–68. 10.1086/648310 19857158

[36] Eppley RW. Temperature and phytoplankton growth in the sea. Fish Bull. 1972;70(4):1063–85.

[37] Norberg J. Biodiversity and ecosystem functioning: A complex adaptive systems approach. Limnology and Oceanography. 2004;49(4part2):1269–77.

[38] Ducklow H. Bacterial production and biomass in the oceans. Microbial ecology of the oceans. 2000;1:85–120.

[39] Kirchman DL. Growth Rates of Microbes in the Oceans. Annual Review of Marine Science. 2016;8(1):285–309. 10.1146/annurev-marine-122414-033938 26195108

[40] del Giorgio PA, Gasol JM. Physiological Structure and Single-Cell Activity in Marine Bacterioplankton. Microbial ecology of the oceans: John Wiley & Sons, Inc.; 2008 p. 243–98.

[41] Thomas MK, Kremer CT, Litchman E. Environment and evolutionary history determine the global biogeography of phytoplankton temperature traits. Global Ecology and Biogeography. 2016;25(1):75–86.

[42] Chandler JW, Lin Y, Gainer PJ, Post AF, Johnson ZI, Zinser ER. Variable but persistent coexistence of Prochlorococcus ecotypes along temperature gradients in the ocean's surface mixed layer. Environmental Microbiology Reports. 2016:n/a-n/a. 10.1111/1758-2229.12378 26743532

[43] Zinser ER, Johnson ZI, Coe A, Karaca E, Veneziano D, Chisholm SW. Influence of light and temperature on Prochlorococcus ecotype distributions in the Atlantic Ocean. Limnology and Oceanography. 2007;52(5):2205–20.

[44] West NJ, Scanlan DJ. Niche-Partitioning of ProchlorococcusPopulations in a Stratified Water Column in the Eastern North Atlantic Ocean. Applied and Environmental Microbiology. 1999;65(6):2585–91. 10347047 10.1128/aem.65.6.2585-2591.1999 PMC91382

[45] Moore LR, Chisholm SW. Photophysiology of the marine cyanobacterium Prochlorococcus: Ecotypic differences among cultured isolates. Limnology and Oceanography. 1999;44(3):628–38.

[46] Rusch DB, Martiny AC, Dupont CL, Halpern AL, Venter JC. Characterization of Prochlorococcus clades from iron-depleted oceanic regions. Proceedings of the National Academy of Sciences. 2010;107(37):16184–9. 10.1073/pnas.1009513107 PMC2941326 20733077

[47] Schmieder R, Edwards R. Quality control and preprocessing of metagenomic datasets. Bioinformatics. 2011;27(6):863–4. 10.1093/bioinformatics/btr026 21278185 PMC3051327

[48] Ondov BD, Treangen TJ, Melsted P, Mallonee AB, Bergman NH, Koren S, et al Mash: fast genome and metagenome distance estimation using MinHash. Genome Biology. 2016;17(1):132 10.1186/s13059-016-0997-x 27323842 PMC4915045

[49] Boyd PW, Rynearson TA, Armstrong EA, Fu F, Hayashi K, Hu Z, et al Marine Phytoplankton Temperature versus Growth Responses from Polar to Tropical Waters–Outcome of a Scientific Community-Wide Study. PLoS ONE. 2013;8(5):e63091 10.1371/journal.pone.0063091 23704890 PMC3660375

[50] Moore LR, Post AF, Rocap G, Chisholm SW. Utilization of different nitrogen sources by the marine cyanobacteria Prochlorococcus and Synechococcus. Limnology and Oceanography. 2002;47(4):989–96.

[51] Moore L, Ostrowski M, Scanlan D, Feren K, Sweetsir T. Ecotypic variation in phosphorus acquisition mechanisms within marine picocyanobacteria. Aquatic Microbial Ecology. 2005.

[52] Kamennaya NA, Post AF. Characterization of Cyanate Metabolism in Marine Synechococcus and Prochlorococcus spp. Applied and Environmental Microbiology. 2011;77(1):291–301. 10.1128/AEM.01272-10 21057026 PMC3019706

[53] Berube PM, Biller SJ, Kent AG, Berta-Thompson JW, Roggensack SE, Roache-Johnson KH, et al Physiology and evolution of nitrate acquisition in Prochlorococcus. ISME J. 2015;9(5):1195–207. 10.1038/ismej.2014.211 25350156 PMC4409163

[54] Leibold MA, Holyoak M, Mouquet N, Amarasekare P, Chase JM, Hoopes MF, et al The metacommunity concept: a framework for multi-scale community ecology. Ecology Letters. 2004;7(7):601–13.

[55] Hellweger FL. Spatially explicit individual-based modeling using a fixed super-individual density. Computers & Geosciences. 2008;34(2):144–52.

[56] Hellweger FL. Carrying photosynthesis genes increases ecological fitness of cyanophage *in silico*. Environmental Microbiology. 2009;11(6):1386–94. 10.1111/j.1462-2920.2009.01866.x 19175665

[57] Hellweger FL. Resonating circadian clocks enhance fitness in cyanobacteria *in silico*. Ecological Modelling. 2010;221(12):1620–9.

[58] Ottesen EA, Young CR, Eppley JM, Ryan JP, Chavez FP, Scholin CA, et al Pattern and synchrony of gene expression among sympatric marine microbial populations. Proceedings of the National Academy of Sciences. 2013;110(6):E488–E97. 10.1073/pnas.1222099110 PMC3568374 23345438

