# Supplementary material for: The Role of Ocean Currents in the Temperature Selection of Plankton: Insights from an Individual-Based Model
Source: PLoS One. 2016 Dec 1;11(12):e0167010. doi: 10.1371/journal.pone.0167010 (PMC5131974; doi:10.1371/journal.pone.0167010)
Supplement: S1 File — This file includes Model description, Selection dynamics, Plankton datasets notes, Additional model results and References. (PDF) [file pone.0167010.s001.pdf]

S1 File

Contents in this file:

S1. Model description
Figs S1 to S4
Tables S1-S3
S2. Selection dynamics
Fig S5
S3. Plankton datasets notes
Fig S6
S4. Additional model results
Fig. S7
S5. References

Other files:

Atlas
Movie

S1. Model description

S1a. Overview

The model simulates microbes using Eulerian and Lagrangian (a.k.a. agent-/individual-based) methods. Both approaches provide essentially the same results in this case (e.g., advective temperature differential, Fig S1), but the Lagrangian method allows for tracking of the temperature history of individuals. Microbes are advected and diffused on a 2°×2° grid using output from a hydrodynamic model. They grow (divide) and die depending on the temperature and local population size, as described in this section.

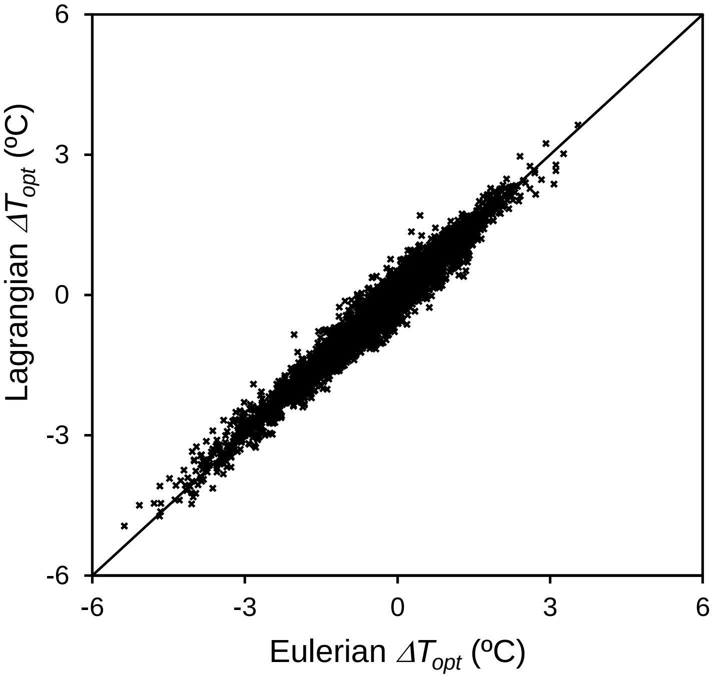

Fig S1. Comparison of Lagrangian and Eulerian approaches. Advective temperature differential predicted for each 2°×2° grid box in the ocean. Population average growth rate = 0.14 d^{-1}. Values are averages over the 31-year simulation period for each grid box.

S1b. Transport

Advection

Cells are transported by advection and diffusion. Advection is based on the Ocean model For the Earth Simulator (OFES), which is based on the MOM3 ocean model (1, 2). OFES is an eddy-resolving ocean model with a horizontal resolution of 1/10º and 54 vertical layers, spanning the oceans from 75ºS to 75ºN and forced by NCEP winds and fluxes. Here, velocity fields averaged

over the top 50m from the last 31 years of the simulation (1980-2010) are used. The data are available as 3-day averages, which is sufficient temporal resolution to accurately capture the mesoscale dynamics (3). Flows are aggregated onto the 2°×2° grid.

This resolution is larger than the width of the Panama Isthmus, which means that in some grid boxes around the isthmus, flow that comes in from the Pacific Ocean could flow into the Atlantic Ocean and vice versa. In order to prevent this unrealistic inter-ocean flux, the Panama Isthmus is artificially closed. Following the method in van Sebille, Beal (4), flow into grid boxes that contain Panama land is set to zero.

Diffusion

A horizontal diffusion coefficient (E) is used to mimic the mixing by sub-mesoscale processes not resolved within the hydrodynamic model. At 200 km effective resolution, the value for diffusion is approximately 500 $m^2\ s^{-1}$ (5), and that value is used here.

Water balance

Time- and spatially-variable horizontal flow rates (Q_X, Q_Y, m^3/s) are obtained from the hydrodynamic model. Flows (and diffusion coefficients) apply to the western and southern interfaces of each 2°×2° grid box. For example, $Q_X(i, j)$ is the flow rate for box (i-1, j) to box (i, j). A vertical (up-/down-welling) flow is calculated based on a water balance, assuming a constant volume:

$$\frac{d(A(i,j)\ H)}{dt} = \frac{d\big(V(i,j)\big)}{dt} = 0 = Q_X(i,j) - Q_X(i+1,j) + Q_Y(i,j) - Q_Y(i,j+1) + Q_Z(i,j) \tag{S1}$$

Where A (m^2) is the area, H (m) is the mixed layer depth, V (m^3) is the volume and Q_X, Q_Y and Q_Z ($m^3\ d^{-1}$) are flow rates. There is no flow across the Southern and Northern boundary and the grid wraps across the Eastern/Western boundary.

Eulerian microbes

For Eulerian tracers, the mass in box (i, j) changes based on horizontal advection (inflow/outflow) and diffusion exchange with the four adjacent boxes, vertical flow, and growth and death.

$$\begin{aligned} V(i,j)\ \frac{dC(i,j)}{dt} = {} & C(i-1,j)\ Q_X(i,j) - C(i,j)\ Q_X(i+1,j) \\ & + C(i,j-1)\ Q_Y(i,j) - C(i,j)\ Q_Y(i,j+1) \\ & + [C(i-1,j) - C(i,j)]\ E_X{}'(i,j) + [C(i+1,j) - C(i,j)]\ E_X{}'(i+1,j) \\ & + [C(i,j-1) - C(i,j)]\ E_Y{}'(i,j) - [C(i,j+1) - C(i,j)]\ E_Y{}'(i,j+1) \\ & + C(i,j)\ Q_Z(i,j) + \big[k_g(i,j) - k_d(i,j)\big]\ C(i,j)\ V(i,j) \end{aligned} \tag{S2}$$

Where C (cells/m^3) is the microbe cell concentration, E_X' and E_Y' (m^3 d^{-1}) are bulk diffusion coefficients ($E' = E\ A_C\ /\ L$, where E (m^2 d^{-1}) is the diffusion coefficient, A_C (m^2) is the cross sectional area (which can be different in the zonal and meridional direction), L (m) is the mixing length, (6), estimated as the distance between the centroids of adjacent boxes). The equation as shown is for the case of positive horizontal flow rates (i.e., northward, eastward). If a flow is negative, the concentration on the other side of the interface is used. The same is true for the case of negative vertical flow rate (i.e., downward flow). If the vertical flow rate is positive, there is no change in mass, because the concentration of microbes below the model layer is assumed to be zero. The growth (k_g, d^{-1}) and death (k_d, d^{-1}) rates change as a function of temperature and population size as described below. The mass balance equation is solved using an explicit numerical integration method. The Eulerian approach is subject to numerical diffusion (6), but comparison to the Lagrangian approach suggests this effect is not substantial in this case (Fig S1).

Lagrangian microbes

For individuals, transport between adjacent boxes is a discrete, stochastic process. The probability of an individual being transported between two adjacent grid boxes in a time step (Δt, d) is based on the flow rate and bulk diffusion coefficient (7-9). For example, the probability of an individual being transported from box (i, j) to box (i+1, j), for the case of positive velocity across that interface, is given below.

$$Probability = \frac{Q_X(i+1,j) + E_X'(i+1,j)}{V(i,j)}\ \Delta t \tag{S3}$$

At each time step and for each individual, a random number is drawn from a standard uniform distribution, and if that number is less than the probability, the cell is transported. Other interfaces are evaluated in the same manner. Division and death are also stochastic processes. For example, the probability of division is defined as follows.

$$Probability = k_g\ \Delta t \tag{S4}$$

The probability of death is calculated in the same manner (using k_d). The growth and death rates are a function of temperature and population size as described in the next section.

S1c. Growth and death rates

The model supports a number of formulations for the effect of temperature and population on growth rate and the death rate. In general, the growth rate (k_g) is a function of a temperature-corrected growth rate ($k_{g,t}$) and a population limitation factor (L_P):

$$k_g = k_{g,t}\, L_P$$

(S5)

The population term on the growth rate controls the population size. In general, the population size will adjust so that the growth rate matches the death rate. That means the average growth rate is controlled by k_d if using Death Model 1 or the *c* parameter if using Death Model 2. However, there are also losses by dilution with upwelling "clean" water (leads to a horizontal net outflow, Eq. S1) in divergence zones. In those areas, the growth rate increases above the death rate.

Effect of temperature on growth rate

Model 1:

The beta formulation (6, 10).

$$k_{g,t} = k_{g,m} \begin{cases} exp\left(-\beta_1 \left(T - T_{opt}\right)^2\right) \; if\; T \leq T_{opt} \\ exp\left(-\beta_2 \left(T_{opt} - T\right)^2\right) \; if\; T > T_{opt} \end{cases}$$

(S6)

This formulation is not used in simulations presented in the paper.

Model 2:

The formulation of Thomas, Kremer (11).

$$k_{g,t} = a\, e^{b\,T} \left[1 - \left(\frac{T - z}{w/2}\right)^2\right]$$

(S7)

a, *b*, *z* and *w* are parameters that control the shape of the growth vs. temperature curves. *z* controls the location of the temperature optimum, but since temperature is also in the exponential term, it is not equal to the optimum temperature. The optimum temperature is obtained numerically. An alternative, numerically-equivalent equation and analytical solution to the optimum temperature was recently presented by Baker, Robinson (12). See Thomas, Kremer (11) for further discussion on these parameters. See the discussion in the main paper on the growth rate vs. temperature curve. The equation is truncated to 0 for negative values. See Fig S2A1. This formulation is used for all simulations except *Prochloroccocus* ecotypes.

Model 3:

A polynomial with lower and upper bounds.

$$k_{g,t} = a_4\,T^4 + a_3\,T^3 + a_2\,T^2 + a_1\,T + a_0 \qquad \text{(S8)}$$

These polynomial functions can cross the x-axis repeatedly. For example, for eMED4 at 5 °C the above equation and parameters in Table S2 yields a positive growth rate. To avoid this, the growth rate is set to zero when the temperature is below a minimum (T_{min}) or above a maximum (T_{max}). These T_{min} and T_{max} parameters characterize the valid range of the equation, not the actual biological temperature range. See Fig S2A2. This formulation is used for *Prochloroccocus* ecotype simulations.

This equation was selected for the *Prochloroccocus* ecotypes from a number of equations, including Eq. S7 and 2nd- to 6th-order polynomials. For each equation, the small sample unbiased Akaike Information Criterion (AIC_c), which quantifies tradeoff between the goodness of fit and complexity, was calculated (13). Specifically, $AIC_c = -2 \ln L + 2K + 2K(K + 1) / (n - K - 1)$, with $\ln L = -(n / 2) \ln (RSS / 2)$, where L is the maximum value of the likelihood function, K is the number of parameters, n is the sample size and RSS is the residual sum of squares. The analysis showed that the 4th-order polynomial is the best model (lowest AIC_c, Table S3).

Effect of population on growth rate

Model 1:

The formulation of Hellweger, van Sebille (14).

$$L_P = \left(1 - \frac{P}{K}\right) \qquad \text{(S9)}$$

P (cells m^{-3}) is the local population size and K (cells m^{-3}) is the carrying capacity. For the Eulerian model, P is the sum of concentrations of all species. For the Lagrangian model, P is the number of individuals (N) divided by the volume (V). See Fig S2B. This formulation is used for all simulations.

Model 2:

The formulation of Thomas, Kremer (11).

$$L_P = \frac{(R_{in} - P)}{(R_{in} - P) + k} \qquad \text{(S10)}$$

This formulation is not used in any of the simulations presented in the paper.

Death rate

Model 1:

Constant death rate (k_d). This formulation is used for *Prochloroccocus* ecotype simulations.

Model 2:

The formulation of Thomas, Kremer (11).

$$k_d = c\ a\ e^{b\ T} \tag{S11}$$

a, b and c are parameters that control the shape of the growth and death vs. temperature curves. See Fig S2C. This formulation is used for all simulations except *Prochloroccocus* ecotypes.

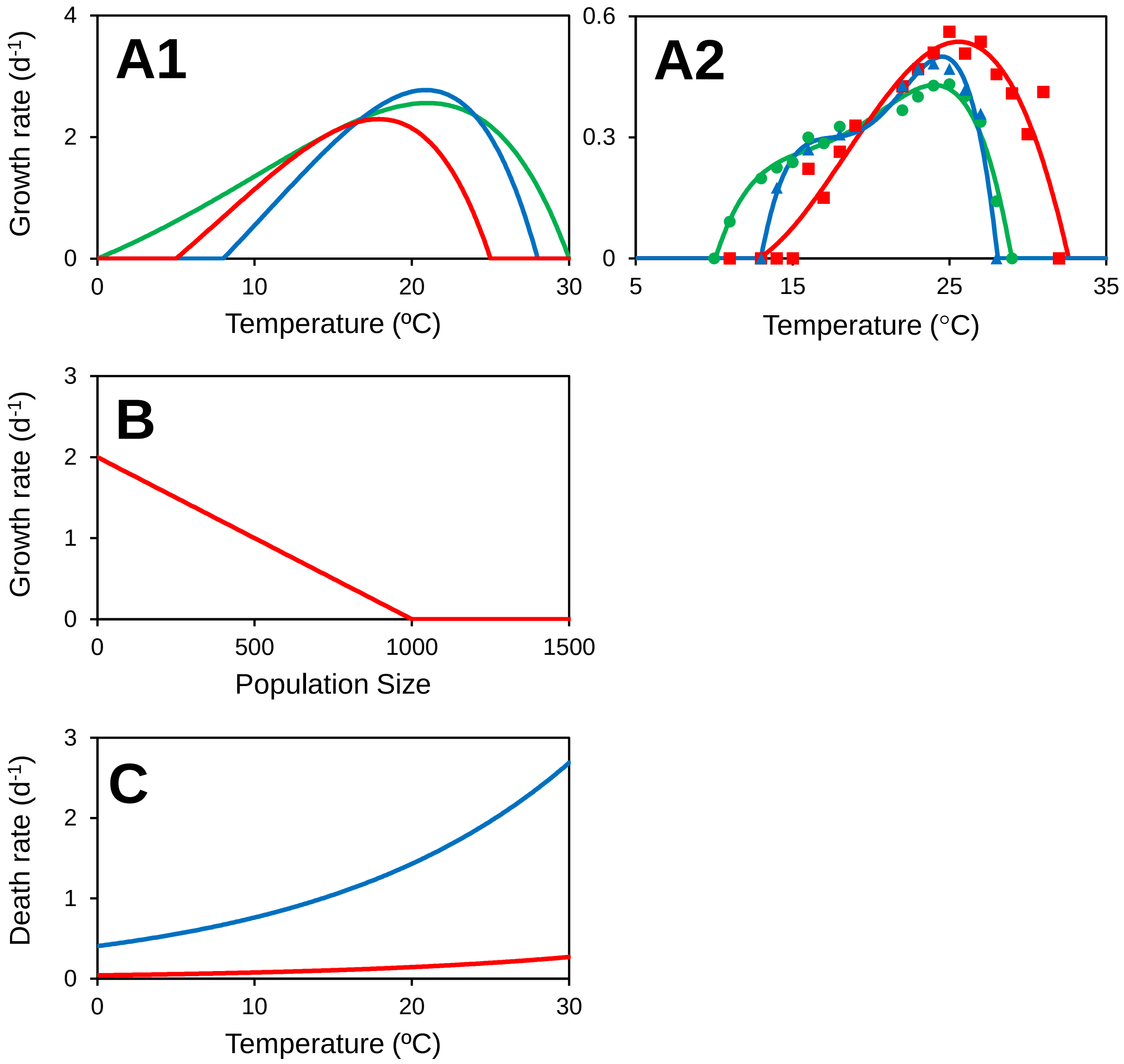

Fig S2. Illustration of growth and death equations. (A) Effect of temperature on growth rate. (A1) Model used for all simulations except *Prochlorococcus* ecotypes. Parameters: z/w = Red: 15/20, Blue: 18/20, Green: 15/30, P = 0. (A2) Model used for *Prochlorococcus* ecotypes simulations. Green: eMED4, Red: eMIT9312, Blue: eNATLA. Data are from Johnson, Zinser (15) and Zinser, Johnson (16). Parameters: See Table S2. (B) Effect of population size on growth rate. Model used for all simulations. Parameters: K = 1,000, k_g = 2 d^{-1} at P = 0. (C) Effect of temperature on death rate. Model used for all simulations except *Prochlorococcus* ecotypes. Parameters: c = Red: 0.05, Blue: 0.5.

S1d. Historical temperature

A comparison of the advective temperature differential (ΔT_{opt}) and historical temperature differential ($\Delta T_{hist}(s) = T_{hist}(s) - T_{loc}$) shows that these quantities are similar.

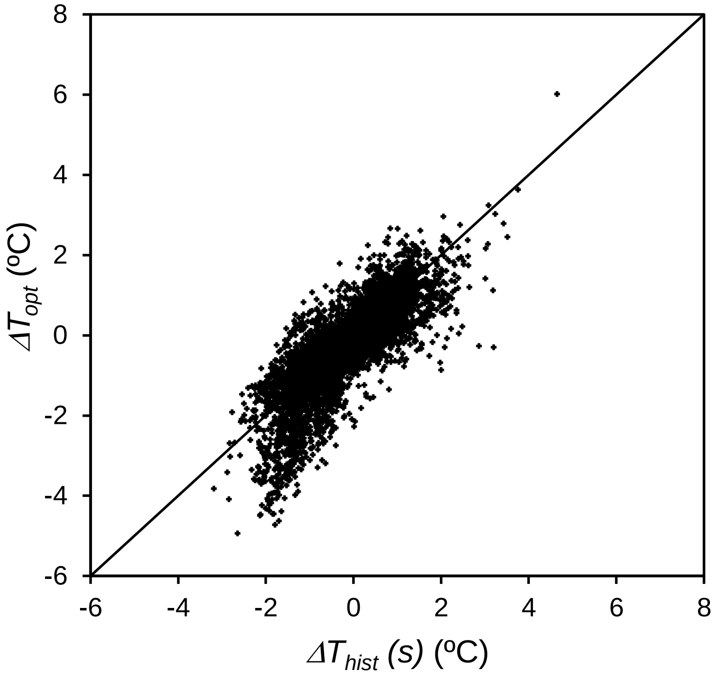

Fig S3. Advective temperature differential (ΔT_{opt}) vs. Historical temperature differential ($\Delta T_{hist}(s)$). Population average growth rate = 0.14 d^{-1}. Values are averages over the 31-year simulation period for each grid box.

S1e. Model implementation and set-up

The model is written in FORTRAN 90 using OpenMP for parallelization with pre-/post-processing in MS Excel with Visual Basic for Applications (VBA). The model code and transport files are available from the corresponding author.

Model parameters are listed in Tables S1&2. Simulations are initialized with a uniform initial total population ($P = K$), distributed evenly across all species. The 31-year model period is preceded by a 1-year spin-up to remove the effect of the initial conditions. A number of species with different temperature parameter (z) were simulated. The temperature parameter was varied from a minimum to a maximum in equal-sized steps, so that the range of T_{opt} values covers the local temperature range. For example, the atlas simulation has 50 species with T_{opt} ranging from -5.2 to 36.2 in 0.8 °C steps. The average number of individuals is controlled by the carrying capacity (K). The number of individuals used in the model is an important parameter, because it controls when a species may become locally extinct under less favorable conditions and thus would not be able to recover when conditions become more favorable. The number of species,

number of individuals and time step vary by simulation. The number of species and individuals were set high enough so that further increases no longer substantially affect the results (e.g., Fig S4). The model is integrated using an explicit finite difference method with a time step a time step (Δt) of 0.3 d. As for the number of species and individuals, the time step was set low enough so that further decreases no longer affect the results. The death parameter (*c*) was assigned to achieve desired average growth rates.

Table S1. Model parameters (All simulations except *Prochloroccocus* ecotypes)

Name	Units	Value	Notes
a	d^{-1}	0.81	= 0.81, (11).
b	$°C^{-1}$	0.0631	= 0.0631, (11).
z	°C	varies	Varies by species. Controls T_{opt}.
w	°C	20	= 10-30, (11)
c	-	varies	= 0.05, 0.5, (11). Varies by simulation. Controls growth rate.
K	cells m^{-3}	varies	Varies by simulation. Controls population size.

Table S2. Model parameters (*Prochlorococcus* ecotypes)

Name	Units	Value All	Value eMED4	Value eMIT9312	Value eNATL2A (a)	Notes
a_4	-	-	-5.929364E-05	-7.173272E-07	-2.189905E-04	(b)
a_3	-	-	+4.349326E-03	-3.166045E-04	+1.735172E-02	(b)
a_2	-	-	-1.175931E-01	+1.897472E-02	-5.085731E-01	(b)
a_1	-	-	+1.408256E+00	-3.009941E-01	+6.557460E+00	(b)
a_0	-	-	-6.087978E+00	+1.426729E+00	-3.116110E+01	(b)
T_{min}	°C	-	10	12	12	(b)
T_{opt}	°C	-	24.0	25.6	24.5	(b)
T_{max}	°C	-	29	33	29	(b)
k_d	d^{-1}	0.17	-	-	-	(c)
K	cells m^{-3}	varies	-	-	-	(d)

(a) This ecotype is not included in the simulation presented in the paper.
(b) See Fig S2A2.
(c) See Section S3.
(d) See Table S1.

Table S3. Model selection (*Prochlorococcus* ecotypes)

**Equation**	***K***	***n***	***RSS***	***AICc***
Eq. S7	4×3	16+16+11	1.1e-1	-222
2nd-order polynomial	3×3	16+16+11	2.8e-1	-193
3rd-order polynomial	4×3	16+16+11	1.4e-1	-211
4th-order polynomial	5×3	16+16+11	7.8e-2	-224*
5th-order polynomial	6×3	16+16+11	6.8e-2	-213
6th-order polynomial	7×3	16+16+11	6.2e-2	-195

(a) The analysis was performed on all three ecotypes simultaneously, so parameters are cumulative.
*Model selected.

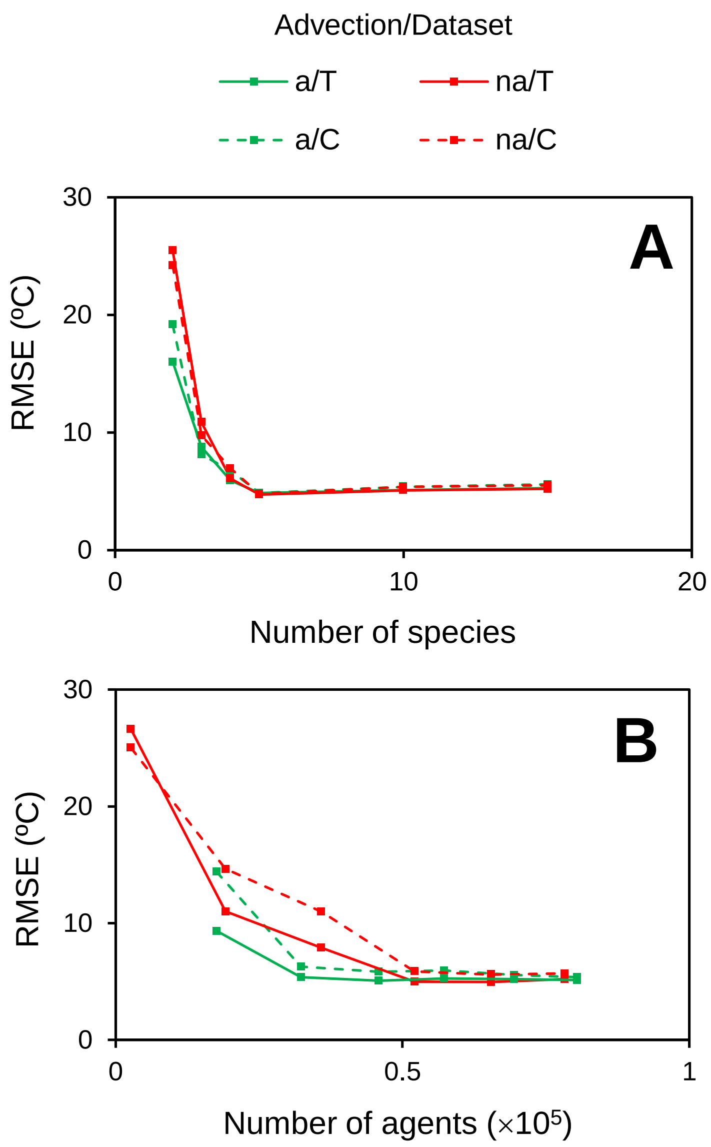

Fig S4. Sensitivity of model results to number of species and individuals. RMSE for advection (a)/no advection (na) and Thomas et al. (T)/Chen et al. (C). (A) Effect of number of species. (B) Effect of number of individuals.

S2. Selection dynamics

The time series of optimum temperatures of the most abundant species can show a bimodal pattern (Fig 2A, Fig S5A). This happens despite the relatively large number of species in the model and is a reflection of the selection dynamics of the system. There are 50 species in this simulation and their growth rates vary in response to the local temperature (Fig S5C). The optimum temperature of the species with the instantaneous highest growth rate smoothly follows the local temperature (Fig S5A, *Topt(a) alt.*). It is higher than the local temperature for reasons discussed in the main paper. However, the optimum temperature of the most abundant species depends on the history of temperature and growth rate. In this case there are only three species that trade this position (Fig S5B). Other species have higher growth rate at times, but not for long enough (average is three weeks) to rise to dominance.

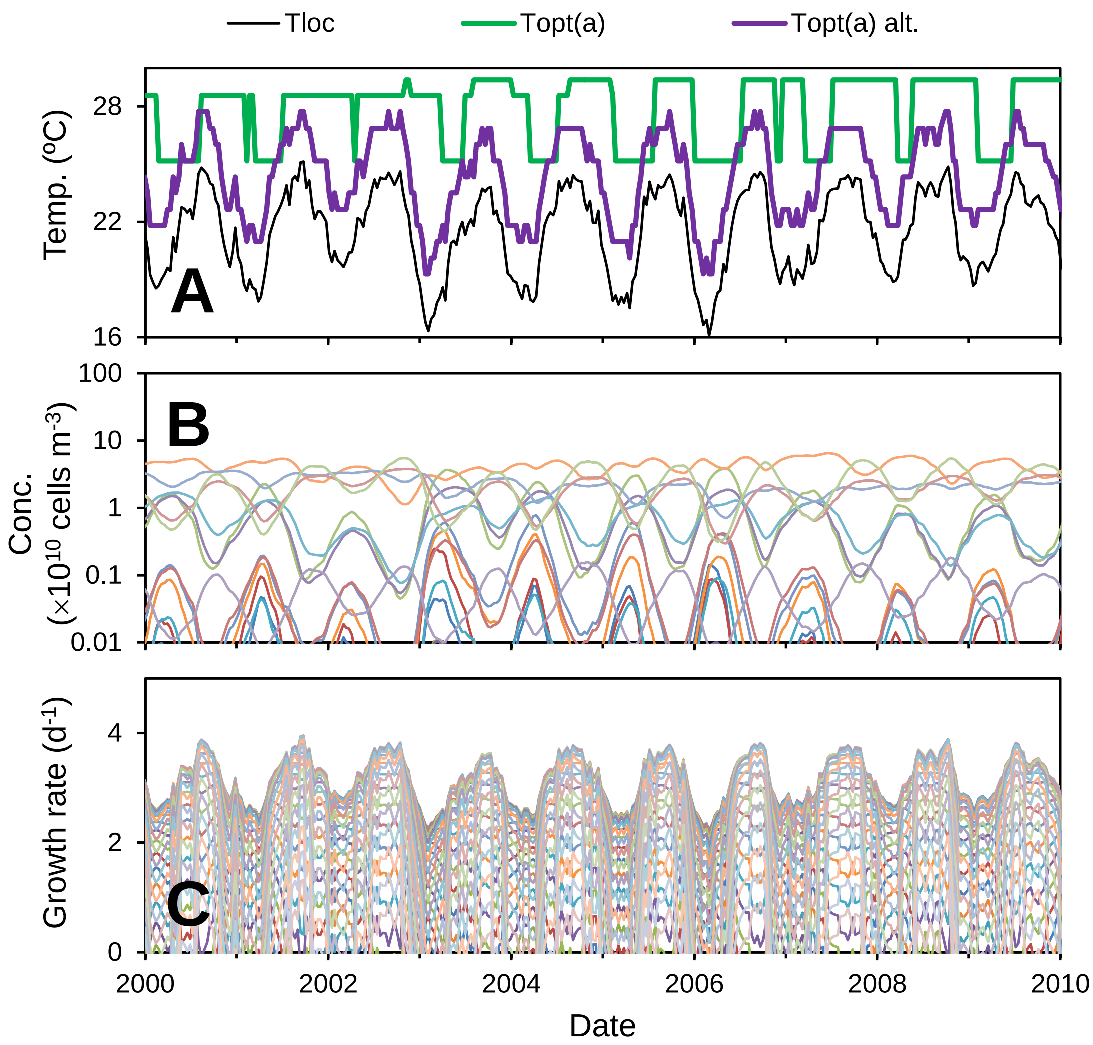

Fig S5. Selection dynamics. (A) Time series of local temperature (T_{loc}), optimum temperature of the most abundant species ($T_{opt}(a)$), and optimum temperature of the species with the highest instantaneous growth rate ($T_{opt}(a)$ *alt.*). (B) Concentration of each species. (C) Growth rate of each species. Same simulation as in Fig 2A, but to increase the resolution the Eulerian concentrations are used.

S3. Plankton datasets notes

S3a. Phytoplankton optimum temperatures

The dataset of Thomas, Kremer (11) includes 194 observations, 153 of which are marine.

The dataset of Chen, Liu (17) includes 513 observations, 222 with coordinates and optimum temperature. Locations for samples ID = 387 and 388 as provided were on land and assumed in

error. We attempted to resolve the issue by referring to the original publication, but were unsuccessful. Those data points are excluded, leaving 220 observations.

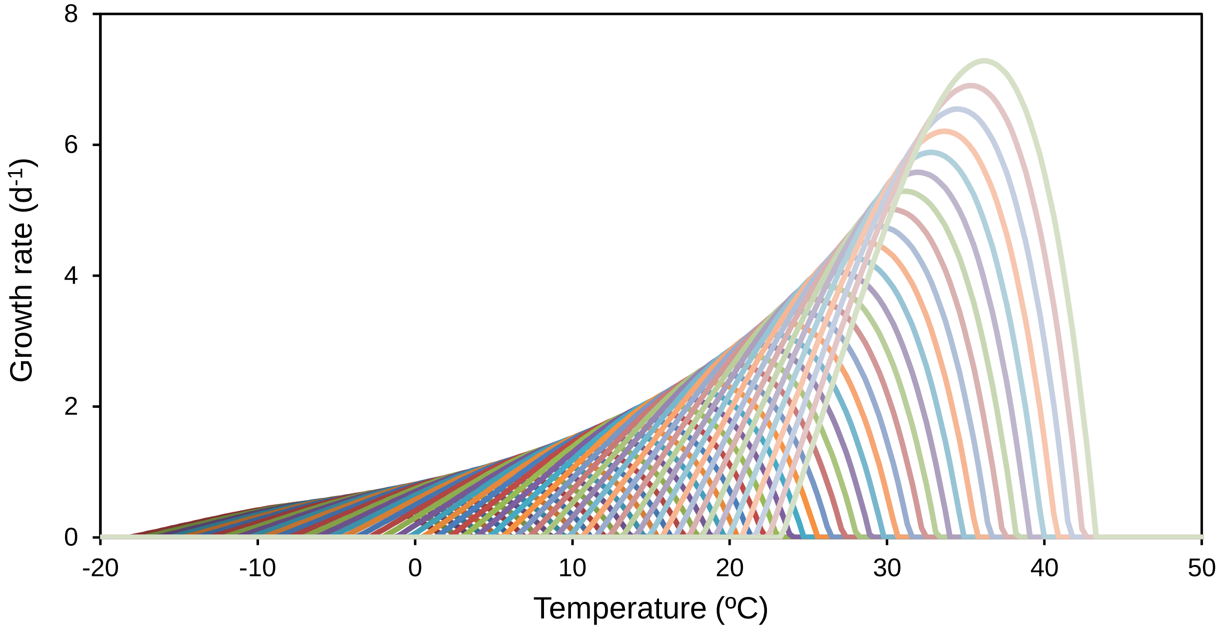

Fig S6. Growth rate vs. temperature function for all 50 species used in the simulation.

S3b. *Prochlorococcus* ecotype ratios

For the comparison presented in Figs 6A and 6B1, the values are the same as those used in the regression in Fig 3 of Chandler, Lin (18) (i.e., all circles). The year of the POWOW1 cruise is outside the range for the model. Model results from 2010 are therefore used. The correction using the atlas and the direct simulation require specification of the average growth rate. Goericke and Welschmeyer (19) found a range of <0.1 to 0.3 d^{-1} for *Prochlorococcus* in the Sargasso Sea. Based on this range, we use a growth rate of 0.18 d^{-1} (average of 0.05 and 0.3). For the direct simulation, the growth rate is controlled by the assigned death rate (k_d), which was set to this value. For the atlas correction, we used the results from the simulation with average growth rate 0.14 d^{-1}. This is lower than 0.18 d^{-1}, but *Prochlorococcus* has a limited latitudinal range (40°N to 40°S, (20) and the growth rate in the model used to develop the atlas generally increases with temperature (see Fig S2A1), so the average growth rate in the said latitudinal range is higher (0.17 d^{-1}) than the global average.

S3c. Metagenome nucleotide divergence

Metagenome sequence sets used to calculate pairwise average nucleotide divergence (AND) measures were quality processed to remove sequence reads determined to be duplicates or that contain known sequencing errors (homopolymer runs and ambiguous bases) using the online bioinformatics tool PRINSEQ (21). The bioinformatic tool Mash was then used to estimate the

pairwise distances. Mash compares all k-mers determined from the provided sequences and calculates a Mash distance between each metagenome pair, a measure found to correlate well with inverse of average nucleotide identity (22). This alignment-free sequence comparison approach is computationally fast and requires less resources than other tools or alignment-based approaches available. The metagenome sequence set MinHash sketch was calculated using the command:

```
mash sketch -u -g 3500 -k 15 -s 50000 -o /mashdata/*.fasta
```

where a k-mer size of 15, a sketch size of 50000 and Bloom filtering of single-copy k-mers was used. Pairwise Mash distances, referred to as AND in our study, were then calculated for all metagenome pairs using the command:

```
mash dist -t /mashdata/AND.msh /mashdata/AND.msh > /mashdata/All_AND
```

S4. Additional model results

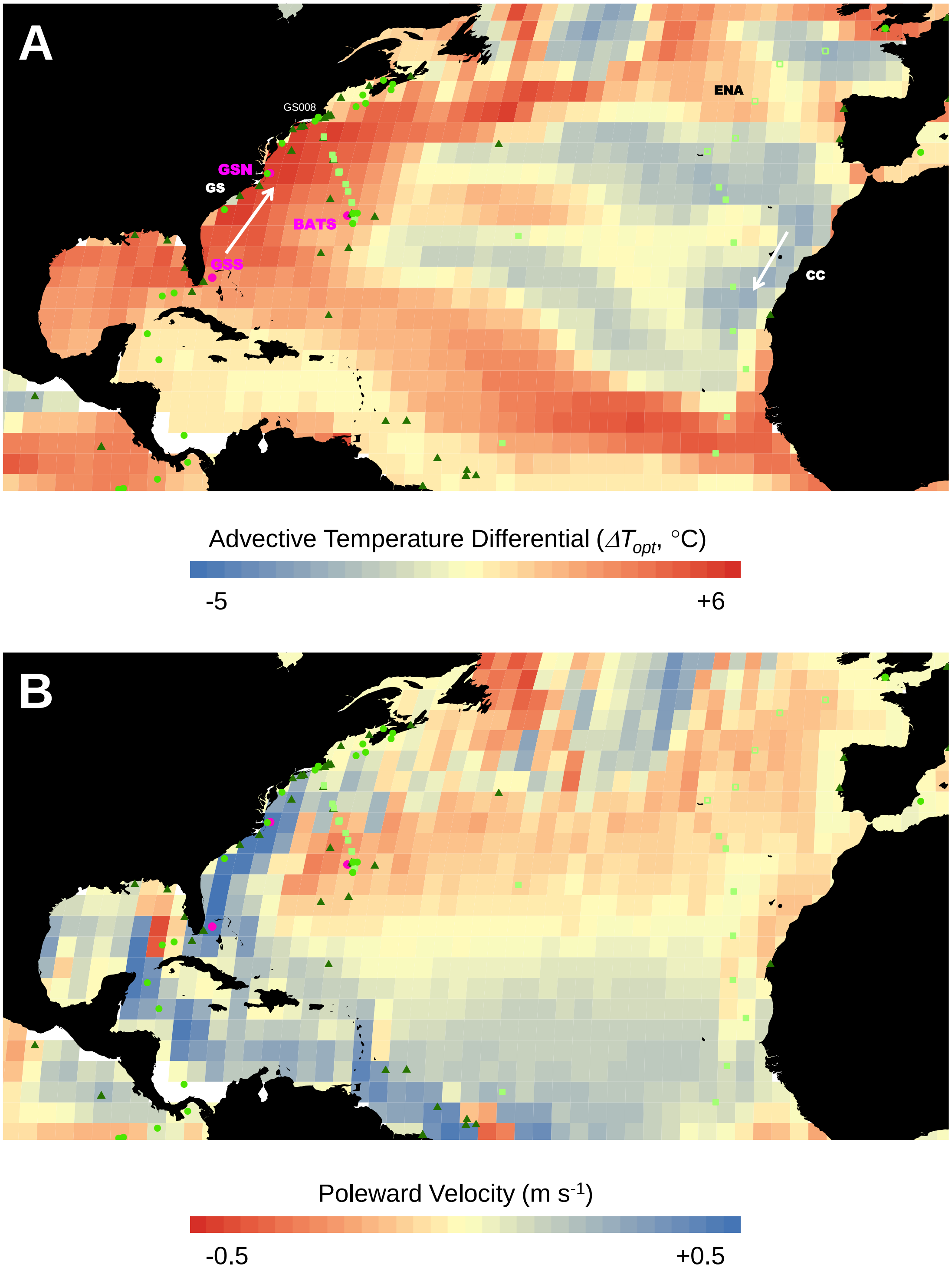

Fig S7. Map of model results, North Atlantic. See legend Fig 3.

S5. References

1. Sasaki H, Nonaka M, Masumoto Y, Sasai Y, Uehara H, Sakuma H. An Eddy-Resolving Hindcast Simulation of the Quasiglobal Ocean from 1950 to 2003 on the Earth Simulator. In: Hamilton K, Ohfuchi W, editors. High Resolution Numerical Modelling of the Atmosphere and Ocean: Springer New York; 2008. p. 157-85.
2. Masumoto Y, Sasaki H, Kagimoto T, Komori N, Ishida A, Sasai Y, et al. A fifty-year eddy-resolving simulation of the world ocean: Preliminary outcomes of OFES (OGCM for the Earth Simulator). J Earth Simulator. 2004;1:35-56.
3. Qin X, van Sebille E, Sen Gupta A. Quantification of errors induced by temporal resolution on Lagrangian particles in an eddy-resolving model. Ocean Modelling. 2014;76(0):20-30.
4. van Sebille E, Beal LM, Johns WE. Advective Time Scales of Agulhas Leakage to the North Atlantic in Surface Drifter Observations and the 3D OFES Model. Journal of Physical Oceanography. 2011;41(5):1026-34.
5. Okubo A. Oceanic diffusion diagrams. Deep Sea Research and Oceanographic Abstracts. 1971;18(8):789-802.
6. Chapra SC. Surface Water-Quality Modeling. Boston: McGraw-Hill; 1997.
7. Hellweger FL. IS IT TIME TO ABANDON THE CHEMISTRY APPROACH TO BIOGEOCHEMISTRY? (AGENT-BASED WATER QUALITY MODELING). Proceedings of the Water Environment Federation. 2007;2007(12):5646-65.
8. Wilkins D, van Sebille E, Rintoul SR, Lauro FM, Cavicchioli R. Advection shapes Southern Ocean microbial assemblages independent of distance and environment effects. Nat Commun. 2013;4.
9. Teske P, Sandoval-Castillo J, Sebille Ev, Waters J, Beheregaray L. On-shelf larval retention limits population connectivity in a coastal broadcast spawner. Marine Ecology Progress Series. 2015;532:1-12.
10. Hellweger FL, Lall U. Modeling the Effect of Algal Dynamics on Arsenic Speciation in Lake Biwa. Environmental Science & Technology. 2004;38(24):6716-23.
11. Thomas MK, Kremer CT, Klausmeier CA, Litchman E. A Global Pattern of Thermal Adaptation in Marine Phytoplankton. Science. 2012;338(6110):1085-8.
12. Baker KG, Robinson CM, Radford DT, McInnes AS, Evenhuis C, Doblin MA. Thermal Performance Curves of Functional Traits Aid Understanding of Thermally Induced Changes in Diatom-Mediated Biogeochemical Fluxes. Frontiers in Marine Science. 2016;3(44).
13. Burnham KP, Anderson DR. Model Selection and Multimodel Inference - A Practical Information-Theoretic Approach. 2nd ed. New York: Springer-Verlag 2002.
14. Hellweger FL, van Sebille E, Fredrick ND. Biogeographic patterns in ocean microbes emerge in a neutral agent-based model. Science. 2014;345(6202):1346-9.
15. Johnson ZI, Zinser ER, Coe A, McNulty NP, Woodward EMS, Chisholm SW. Niche Partitioning Among Prochlorococcus Ecotypes Along Ocean-Scale Environmental Gradients. Science. 2006;311(5768):1737-40.
16. Zinser ER, Johnson ZI, Coe A, Karaca E, Veneziano D, Chisholm SW. Influence of light and temperature on Prochlorococcus ecotype distributions in the Atlantic Ocean. Limnology and Oceanography. 2007;52(5):2205-20.
17. Chen B, Liu H, Huang B, Wang J. Temperature effects on the growth rate of marine picoplankton. Marine Ecology Progress Series. 2014;505:37-47.
18. Chandler JW, Lin Y, Gainer PJ, Post AF, Johnson ZI, Zinser ER. Variable but persistent coexistence of Prochlorococcus ecotypes along temperature gradients in the ocean's surface mixed layer. Environmental Microbiology Reports. 2016:n/a-n/a.

19. Goericke R, Welschmeyer NA. The marine prochlorophyte Prochlorococcus contributes significantly to phytoplankton biomass and primary production in the Sargasso Sea. Deep Sea Research Part I: Oceanographic Research Papers. 1993;40(11–12):2283-94.
20. Partensky F, Hess WR, Vaulot D. Prochlorococcus, a Marine Photosynthetic Prokaryote of Global Significance. Microbiology and Molecular Biology Reviews. 1999;63(1):106-27.
21. Schmieder R, Edwards R. Quality control and preprocessing of metagenomic datasets. Bioinformatics. 2011;27(6):863-4.
22. Ondov BD, Treangen TJ, Melsted P, Mallonee AB, Bergman NH, Koren S, et al. Mash: fast genome and metagenome distance estimation using MinHash. Genome Biology. 2016;17(1):132.
