# Supplementary material for: The Role of Ocean Currents in the Temperature Selection of Plankton: Insights from an Individual-Based Model
Source: PLoS One. 2016 Dec 1;11(12):e0167010. doi: 10.1371/journal.pone.0167010 (PMC5131974; doi:10.1371/journal.pone.0167010)
Supplement: S2 File — The atlas is presented as maps in PDF format (ATLASMAPS) and an MS Excel book (ATLASTABLE) and accompanying ESRI shapefile (ATLASGIS). The atlas includes results from simulations with different average growth rates. Columns/attributes are named using the variables used throughout the paper. The average growth rate is appended to the column name. For example, TOPTA028 is Topt(a) from the simulation with average growth rate of 0.28 d-1. (ZIP) [file pone.0167010.s002.zip › ATLASMAPS.pdf]

Local temperature (T_{LOC}, °C)

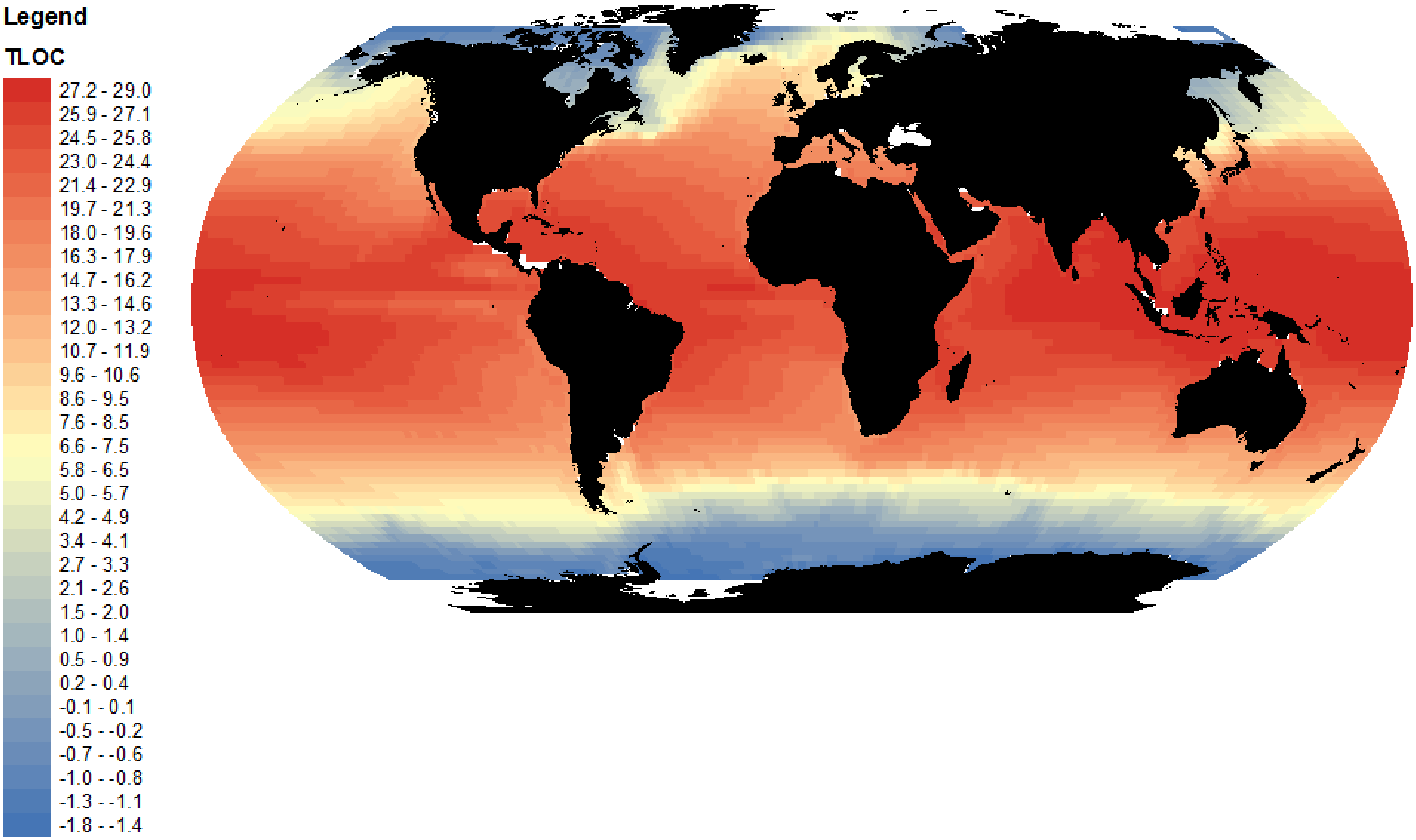

TLOC

[IAM, 10.0.gt.a, 9/4/2015, Run A2, Cip]

Optimum temperature, with advection ($T_{LOC}(a)$, °C)
Pop. ave. grow rate = 0.07/d

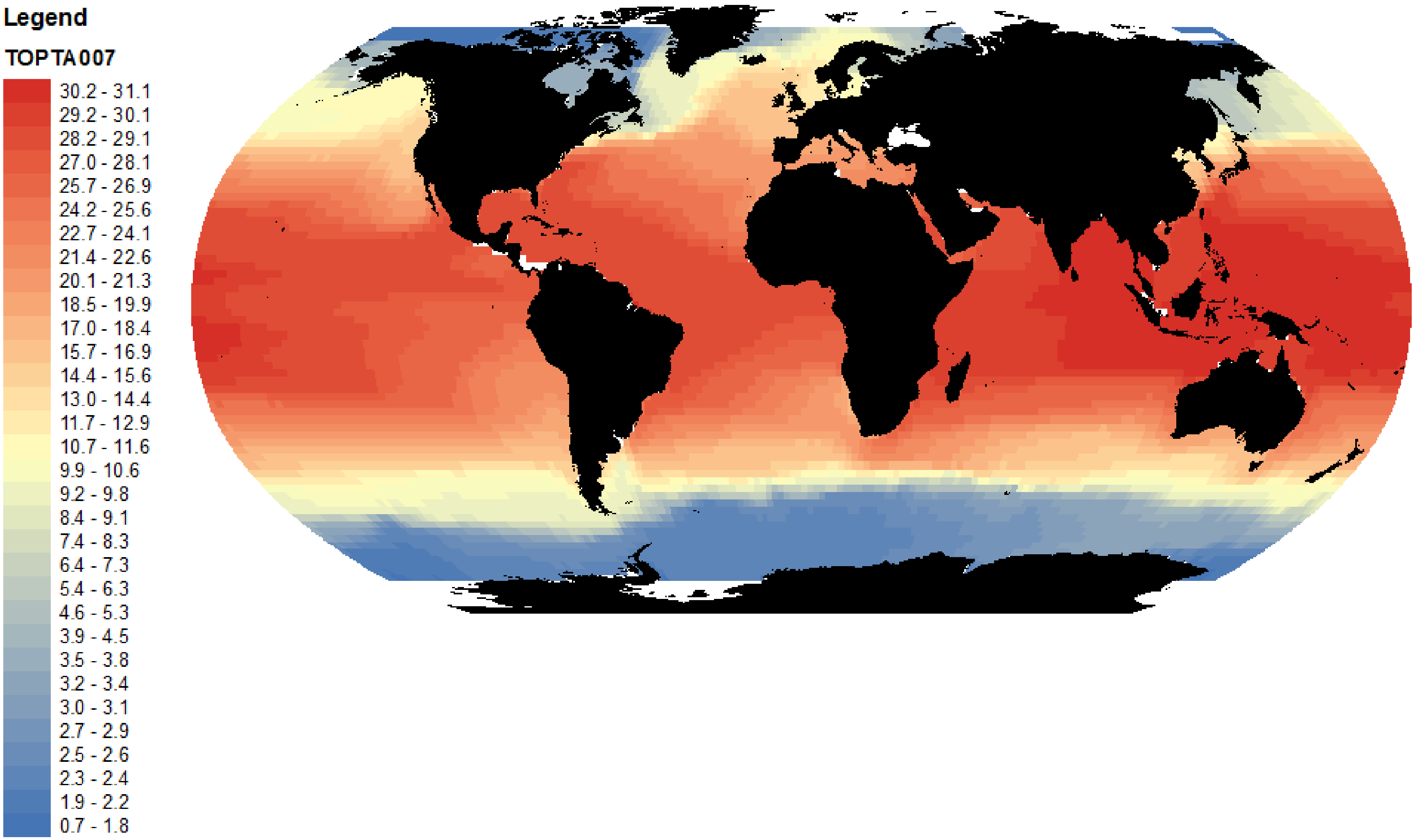

TOPTA007

[IAM, 10.0.gt.a, 9/4/2015, Run A2, Cip]

Optimum temperature, without advection (T_{LOC}*(na)*, °C)
Pop. ave. grow rate = 0.07/d

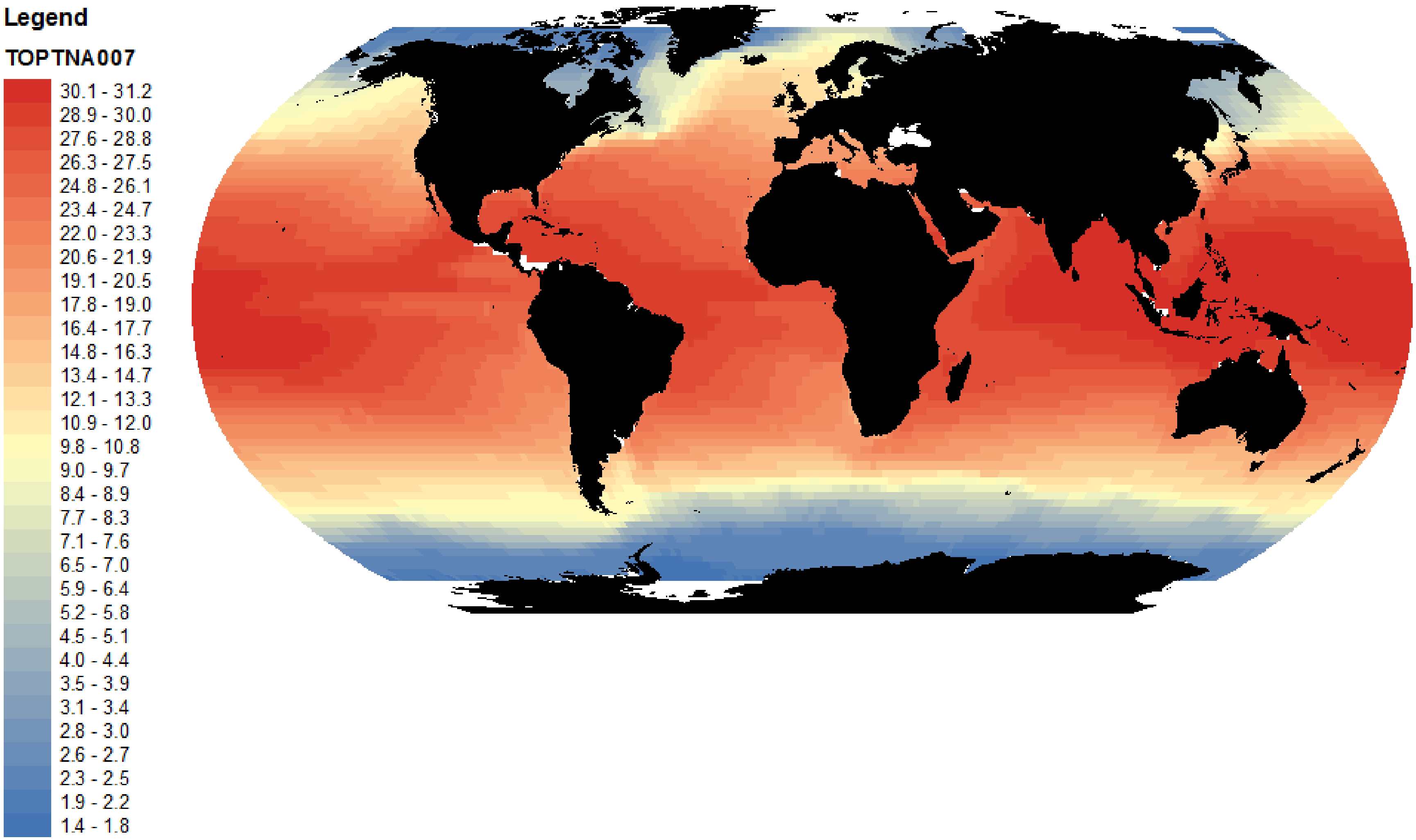

TOPTNA007

[IAM, 10.0.gt.a, 9/4/2015, Run A2, Cip]

Advective temperature differential (ΔT_{OPT}, °C)
Pop. ave. grow rate = 0.07/d

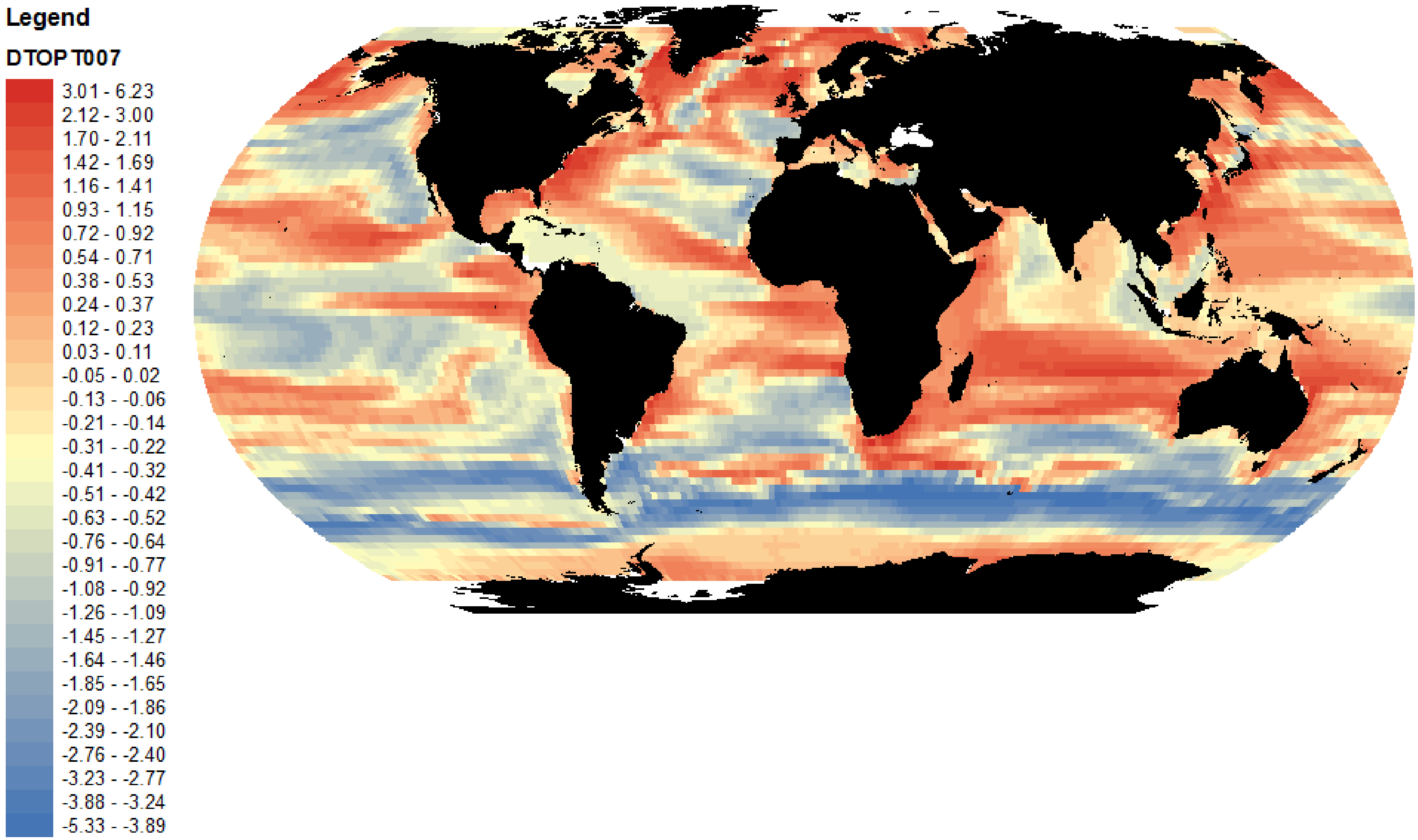

DTOPT007

[IAM, 10.0.gt.a, 9/4/2015, Run A2, Cip]

Optimum temperature, with advection ($T_{LOC}(a)$, °C)
Pop. ave. grow rate = 0.14/d

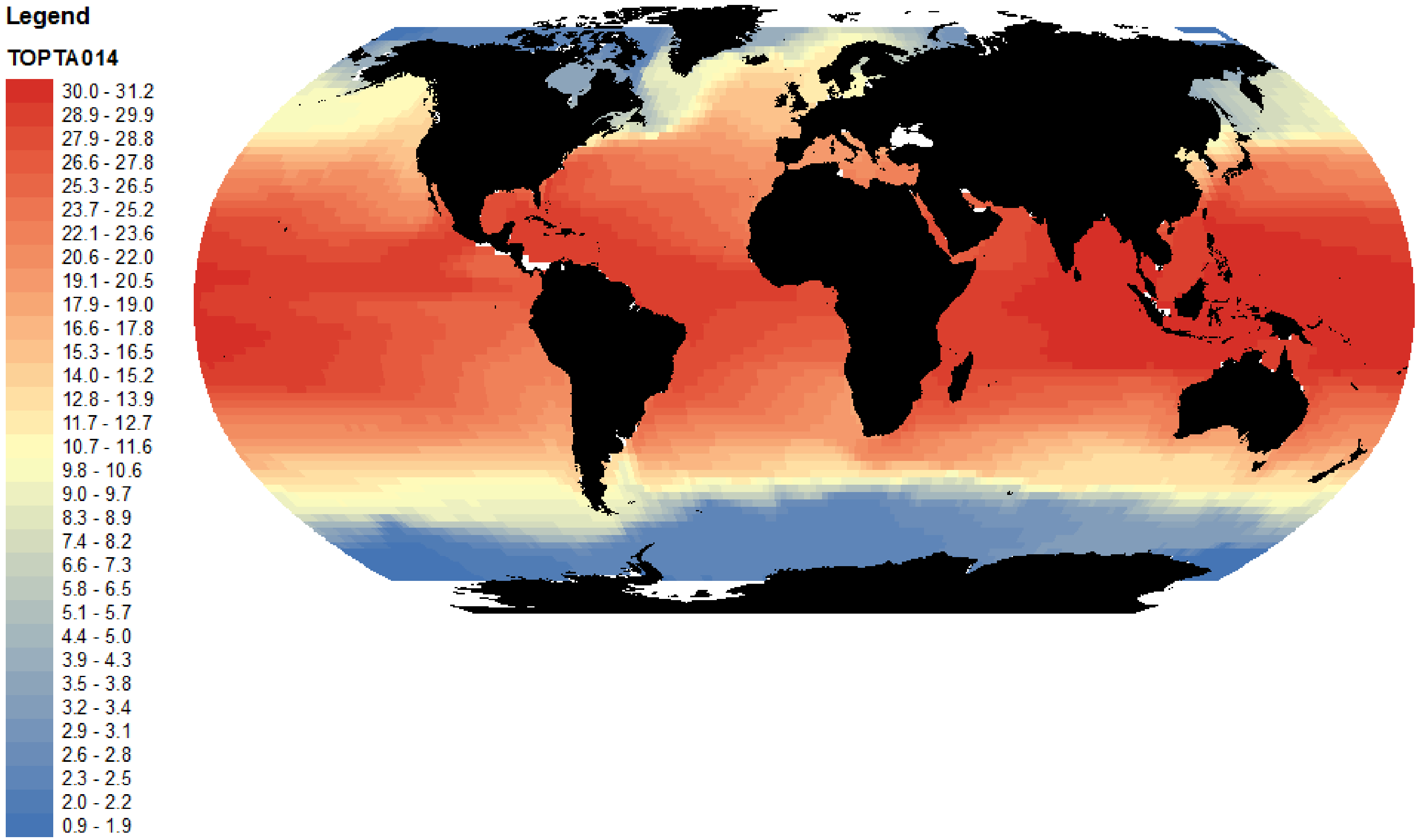

TOPTA014

[IAM, 10.0.gt.a, 9/4/2015, Run A2, Cip]

Optimum temperature, without advection (T_{LOC}*(na)*, °C)
Pop. ave. grow rate = 0.14/d

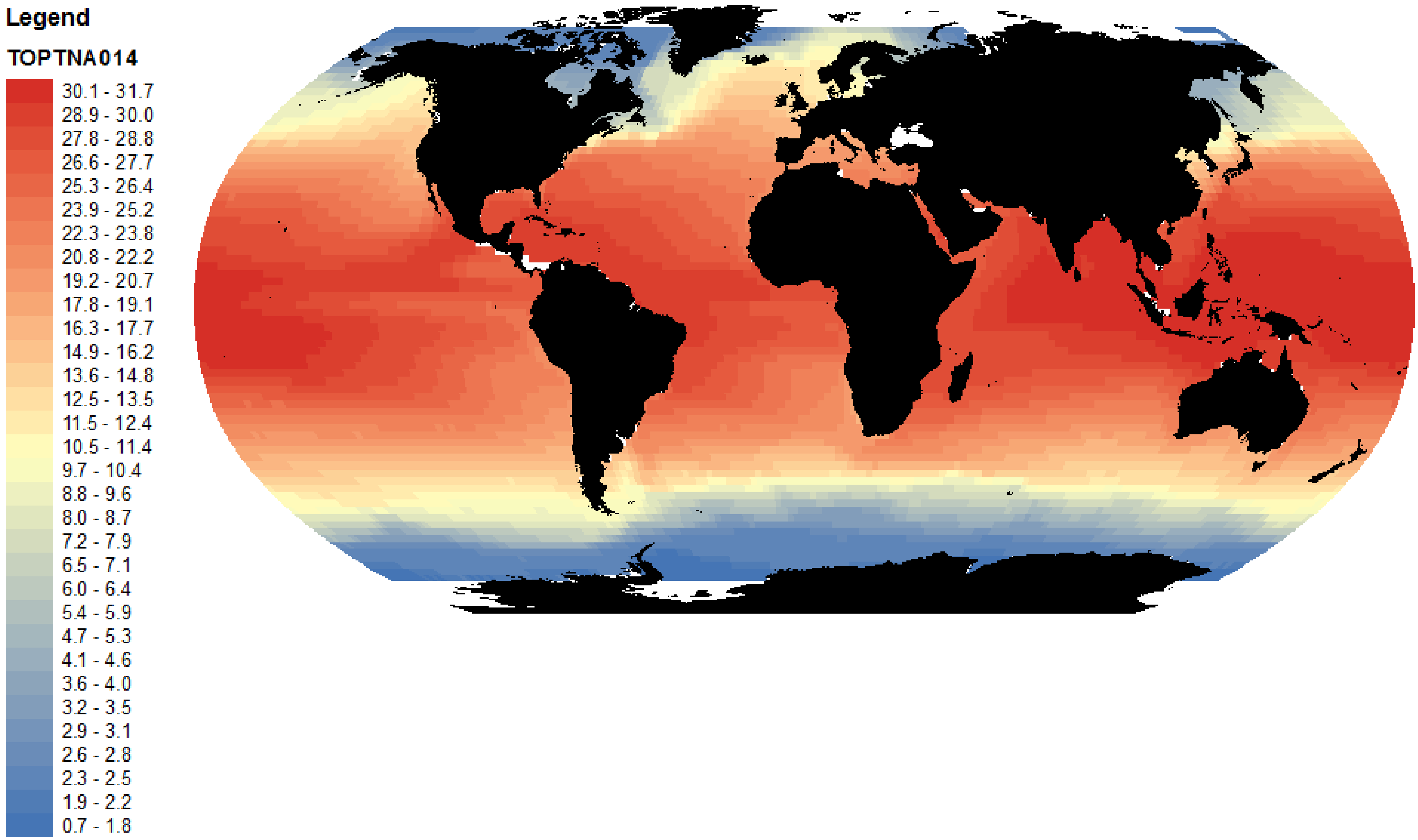

TOPTNA014

[IAM, 10.0.gt.a, 9/4/2015, Run A2, Cip]

Advective temperature differential (ΔT_{OPT}, °C)
Pop. ave. grow rate = 0.14/d

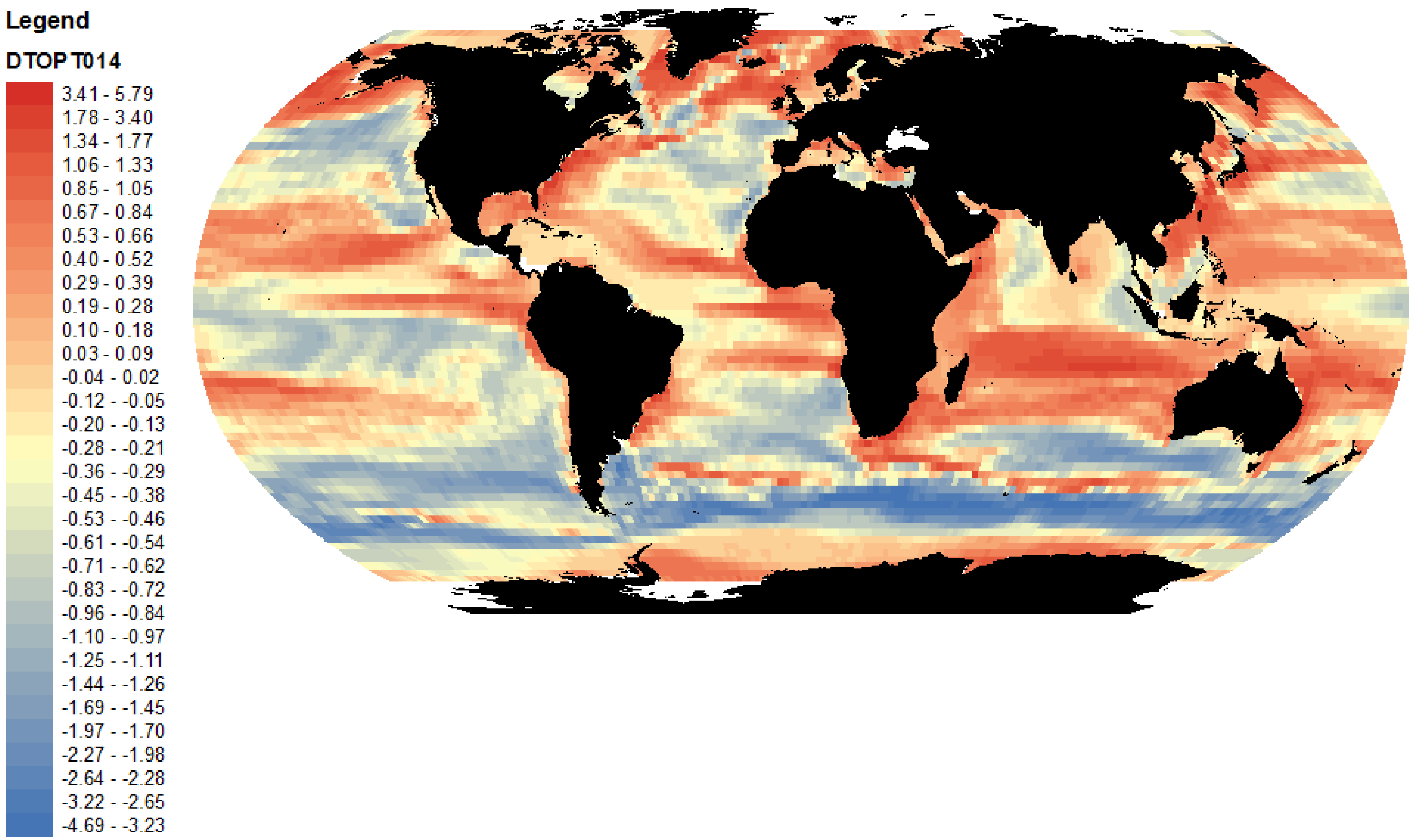

DTOPT014

[IAM, 10.0.gt.a, 9/4/2015, Run A2, Cip]

Optimum temperature, with advection ($T_{LOC}(a)$, °C)
Pop. ave. grow rate = 0.39/d

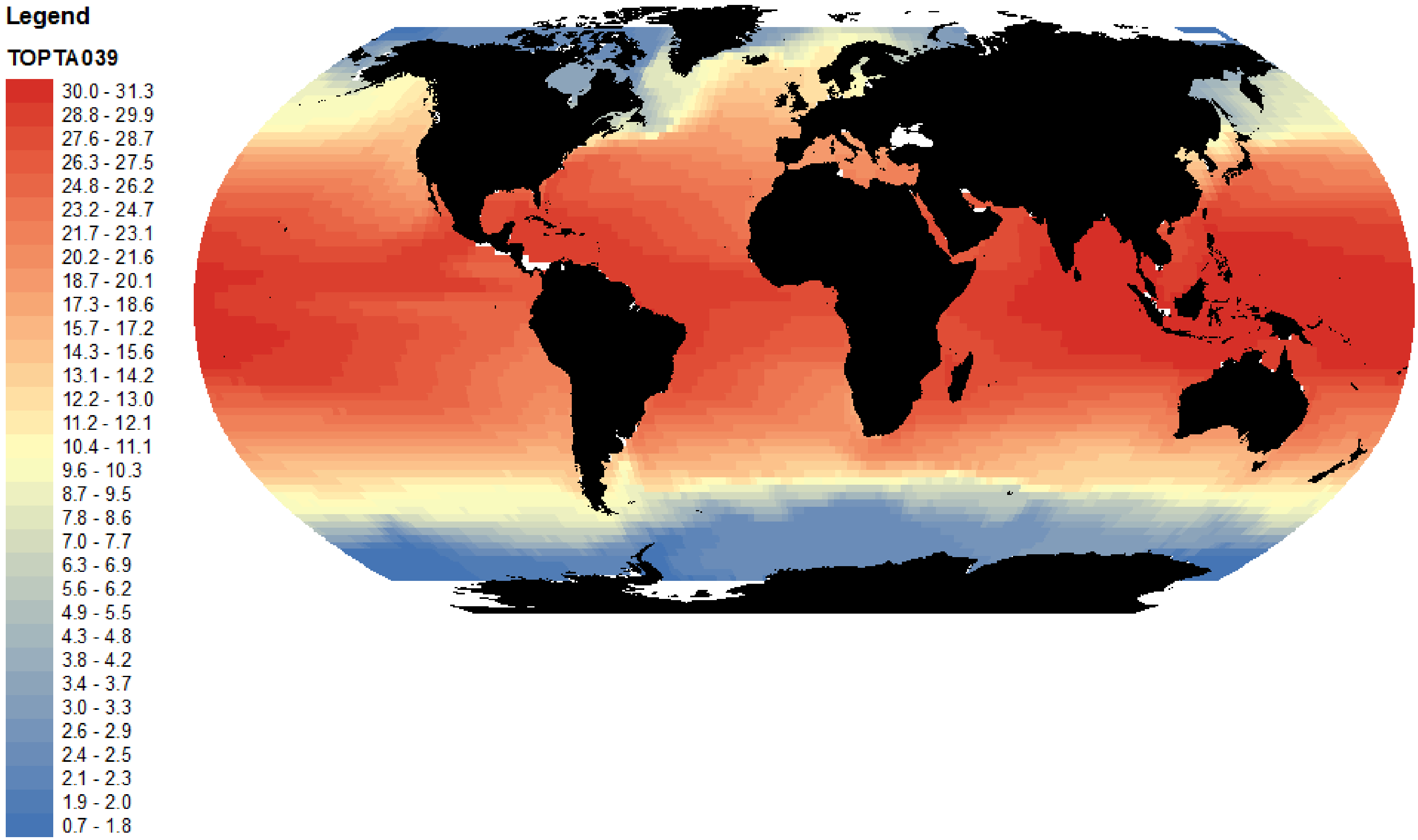

TOPTA039

[IAM, 10.0.gt.a, 9/4/2015, Run A2, Cip]

Optimum temperature, without advection (T_{LOC}*(na)*, °C)
Pop. ave. grow rate = 0.39/d

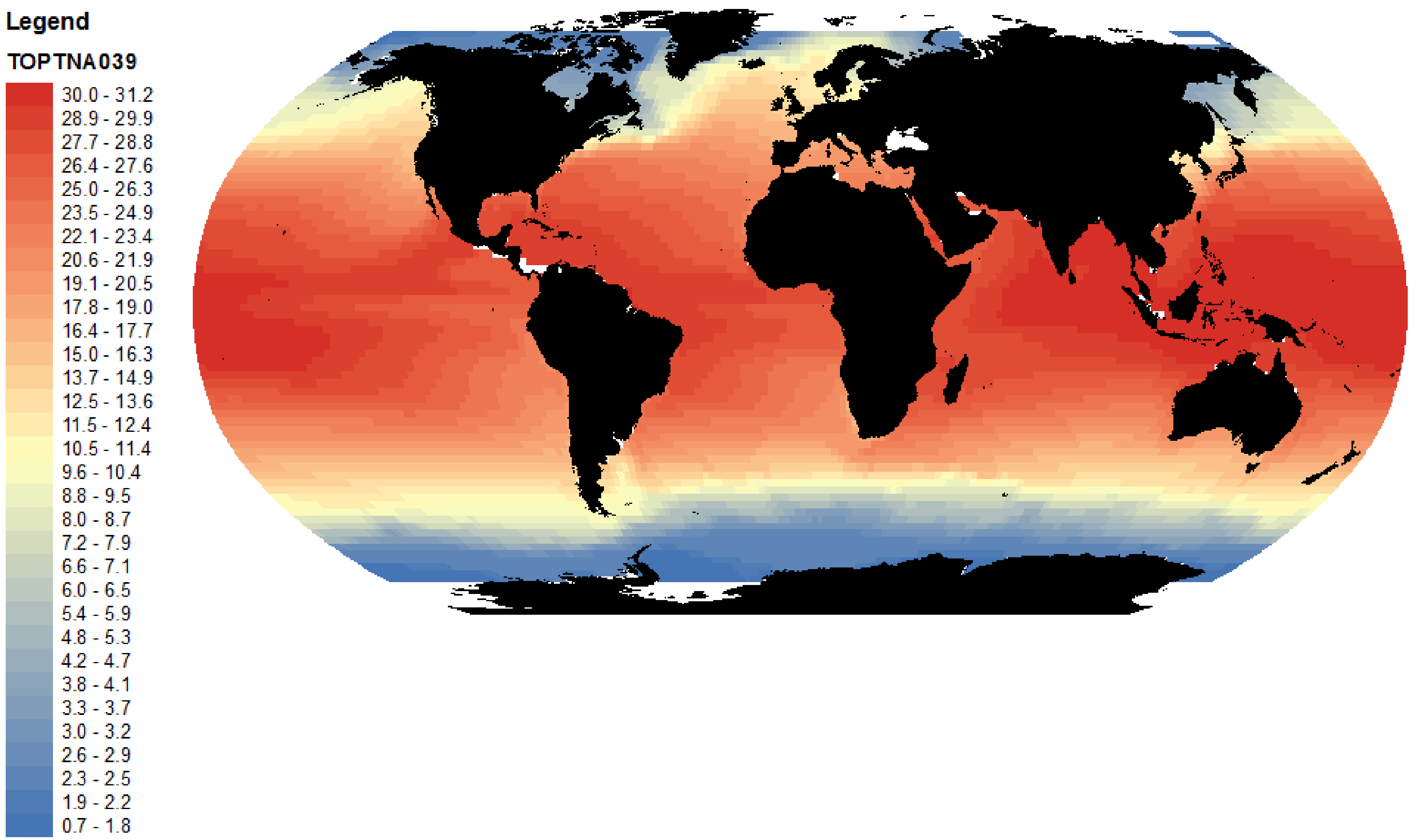

TOPTNA039

[IAM, 10.0.gt.a, 9/4/2015, Run A2, Cip]

Advective temperature differential (ΔT_{OPT}, °C)
Pop. ave. grow rate = 0.39/d

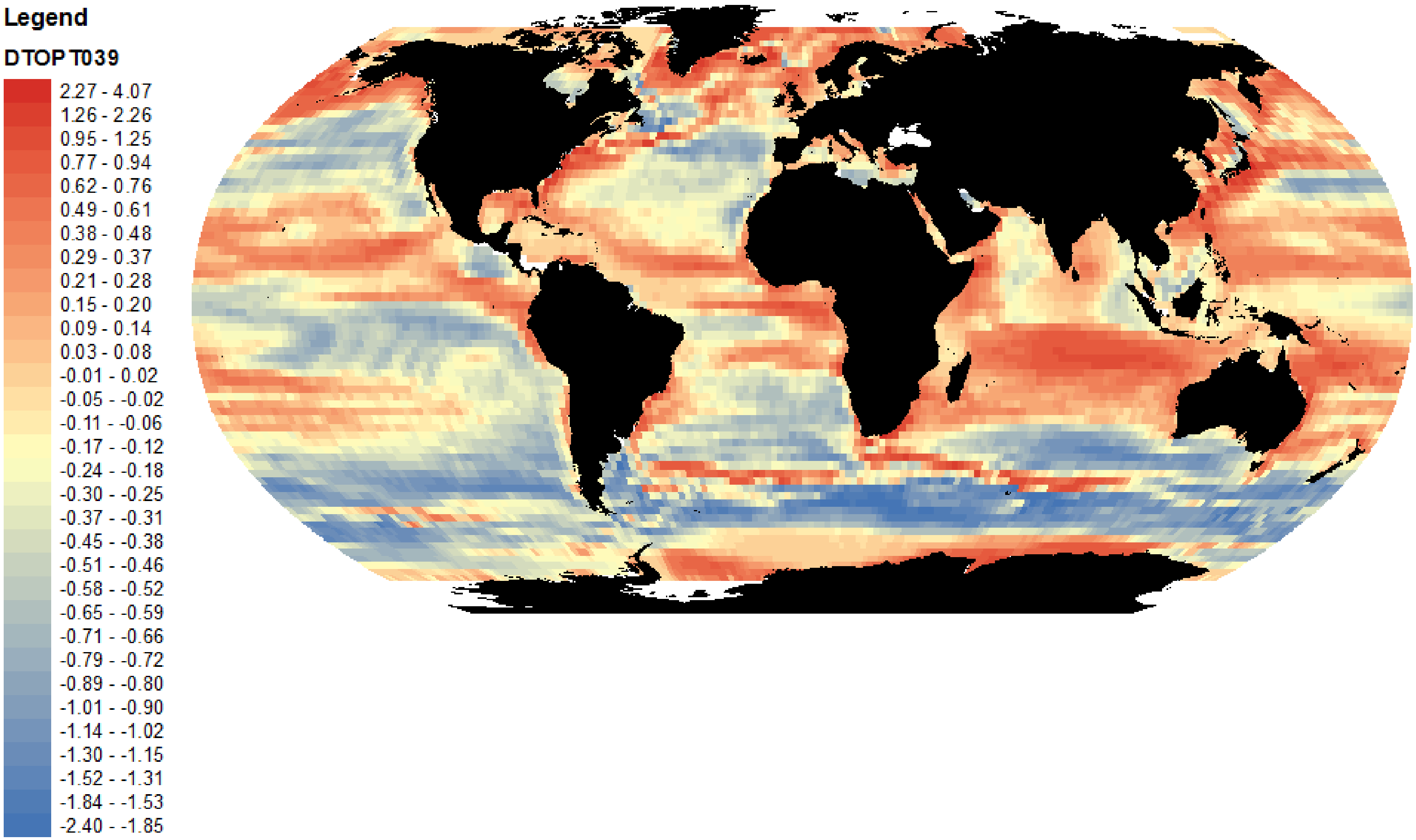

DTOPT039

[IAM, 10.0.gt.a, 9/4/2015, Run A2, Cip]

Optimum temperature, with advection ($T_{LOC}(a)$, °C)
Pop. ave. grow rate = 0.65/d

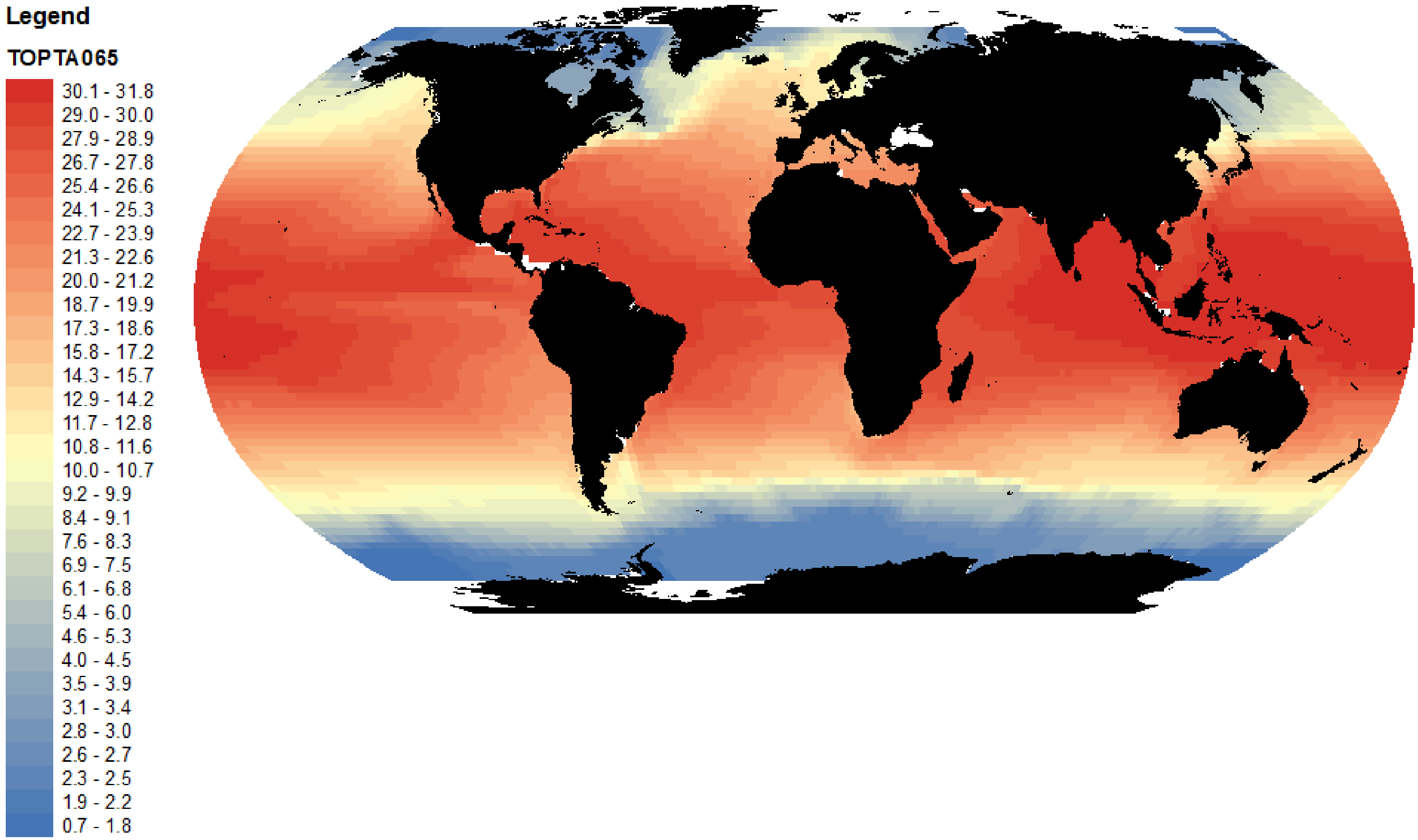

TOPTA065

[IAM, 10.0.gt.a, 9/4/2015, Run A2, Cip]

Optimum temperature, without advection ($T_{LOC}(na)$, °C)
Pop. ave. grow rate = 0.65/d

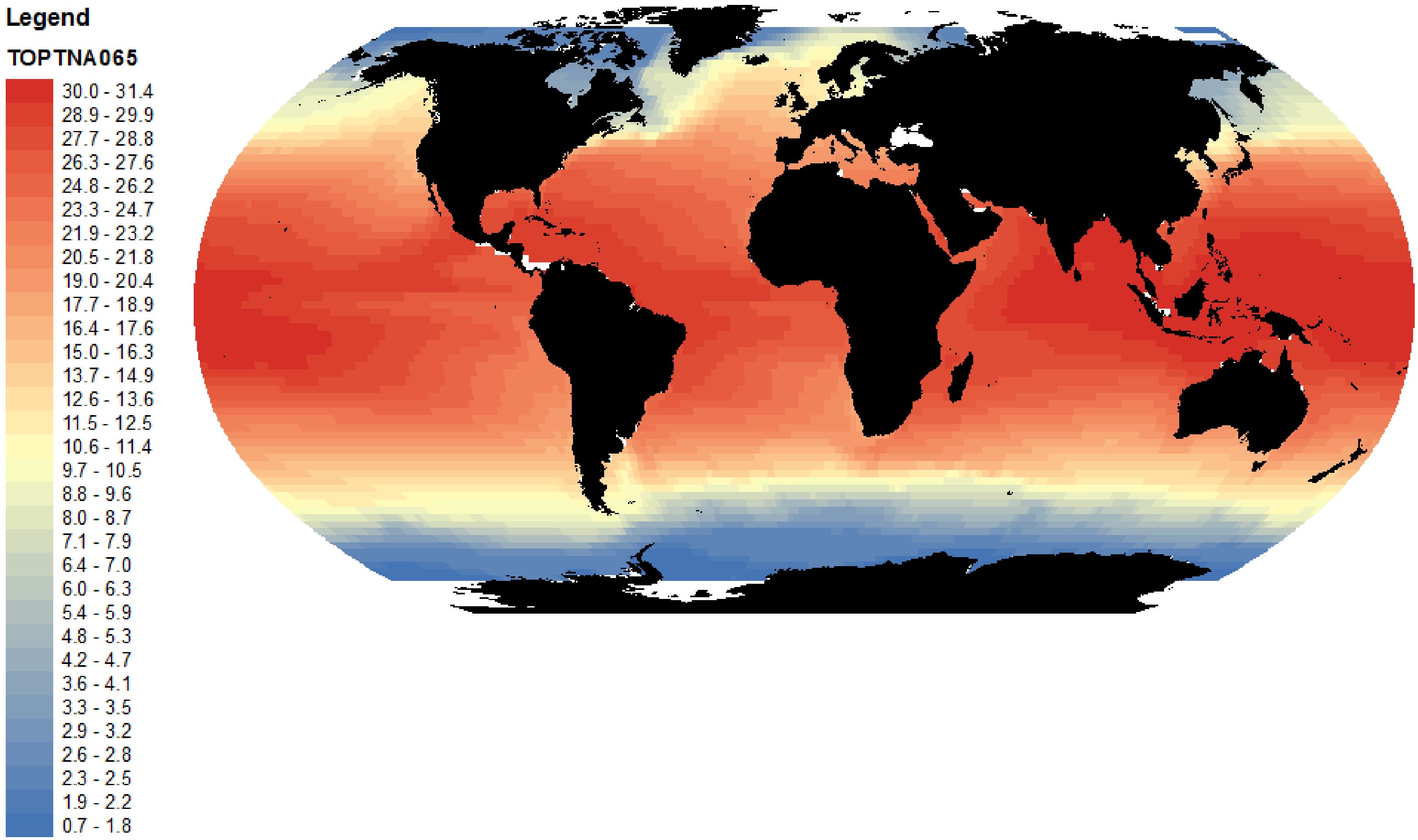

TOPTNA065

[IAM, 10.0.gt.a, 9/4/2015, Run A2, Cip]

Advective temperature differential (ΔT_{OPT}, °C)
Pop. ave. grow rate = 0.65/d

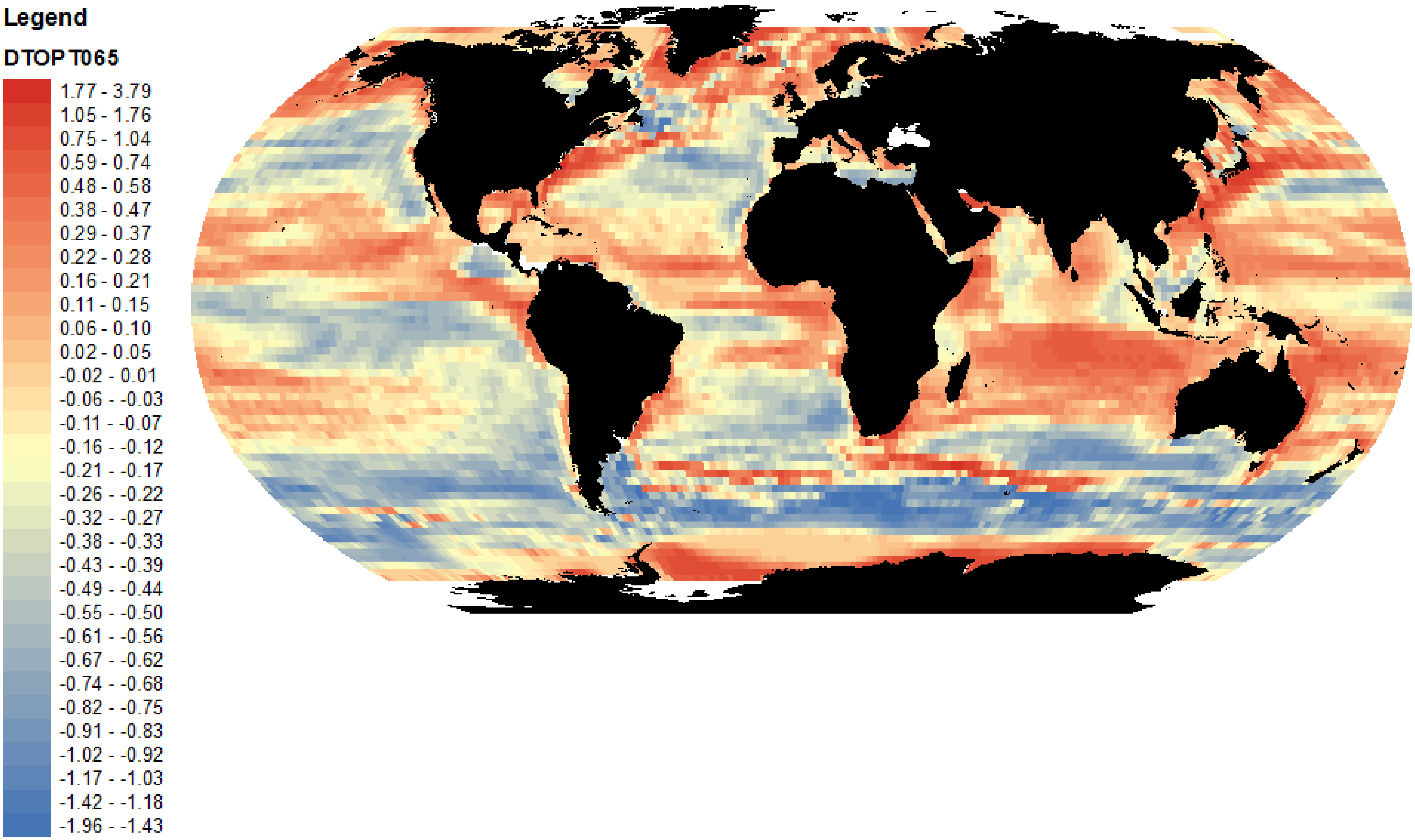

DTOPT065

[IAM, 10.0.gt.a, 9/4/2015, Run A2, Cip]

Optimum temperature, with advection ($T_{LOC}(a)$, °C)
Pop. ave. grow rate = 0.97/d

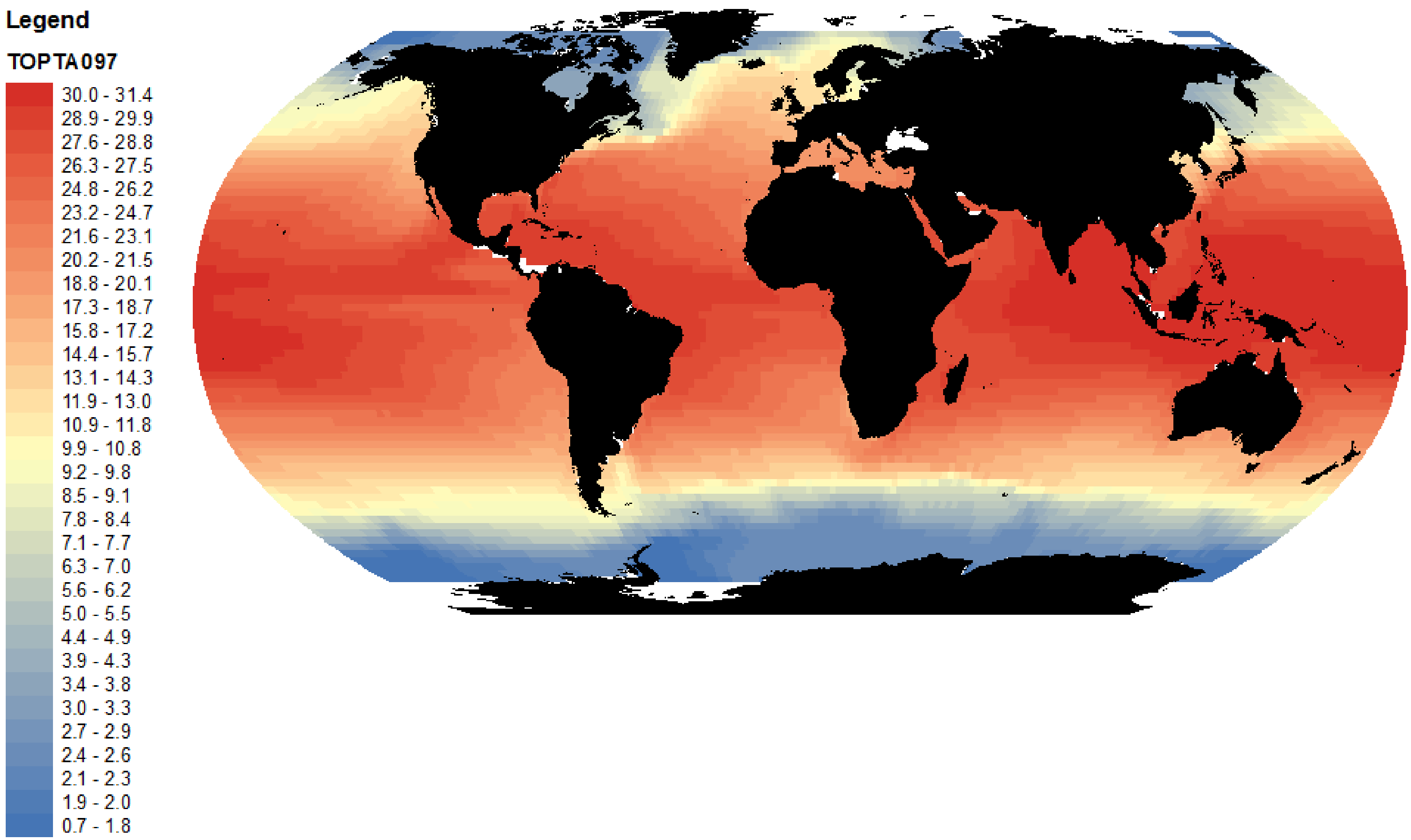

TOPTA097

[IAM, 10.0.gt.a, 9/4/2015, Run A2, Cip]

Optimum temperature, without advection ($T_{LOC}(na)$, °C)
Pop. ave. grow rate = 0.97/d

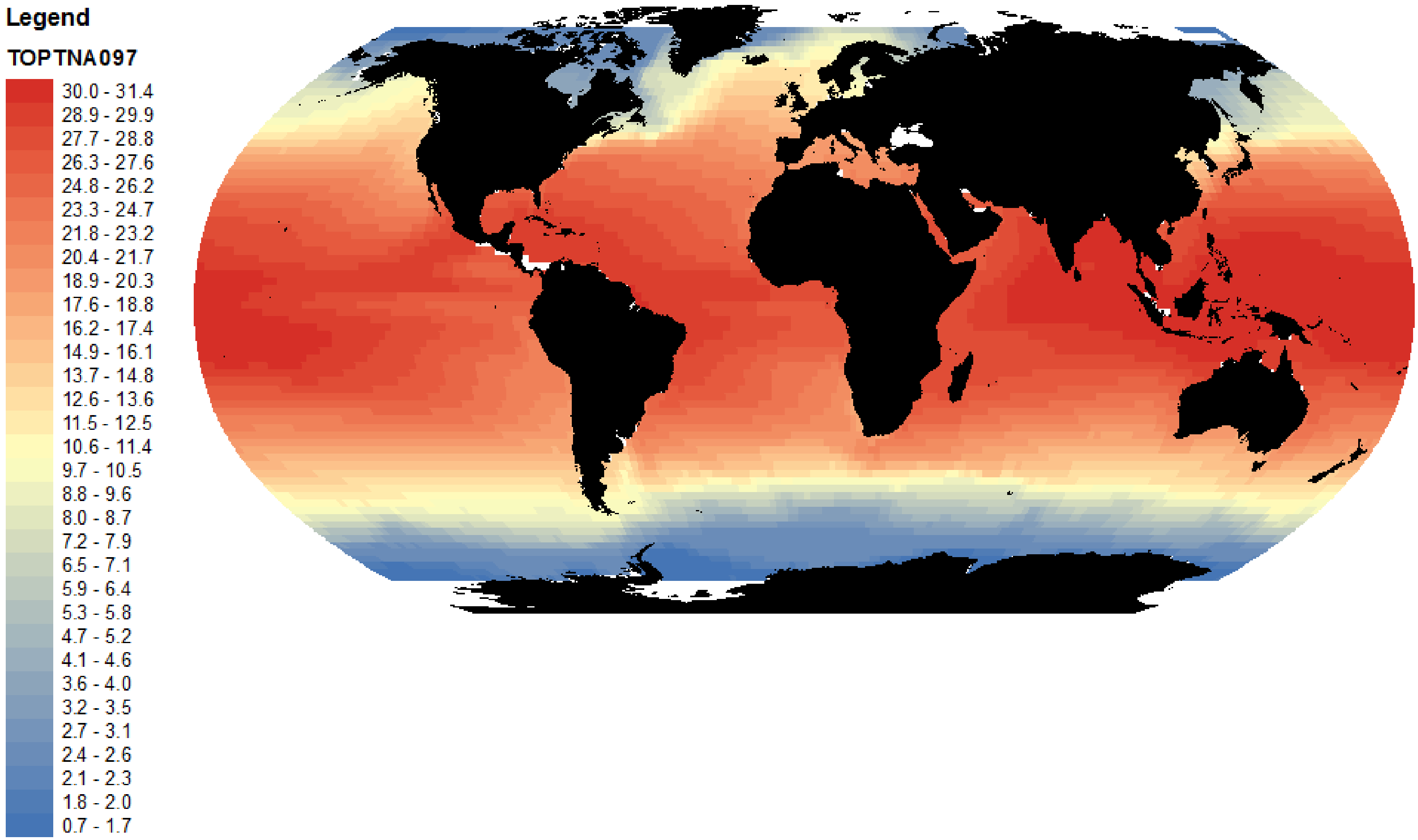

TOPTNA097

[IAM, 10.0.gt.a, 9/4/2015, Run A2, Cip]

Advective temperature differential (ΔT_{OPT}, °C)
Pop. ave. grow rate = 0.97/d

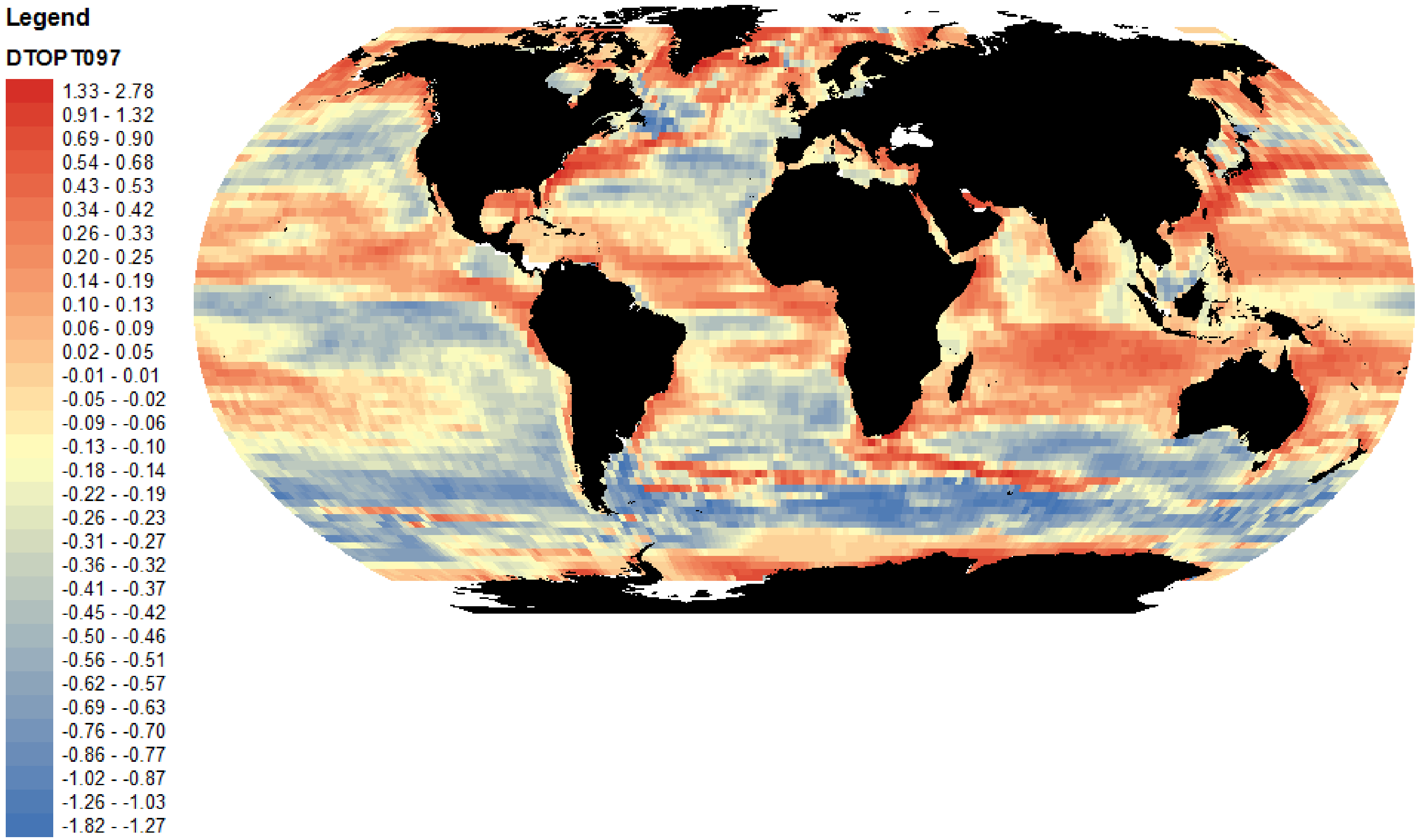

DTOPT097

[IAM, 10.0.gt.a, 9/4/2015, Run A2, Cip]

Optimum temperature, with advection ($T_{LOC}(a)$, °C)
Pop. ave. grow rate = 1.29/d

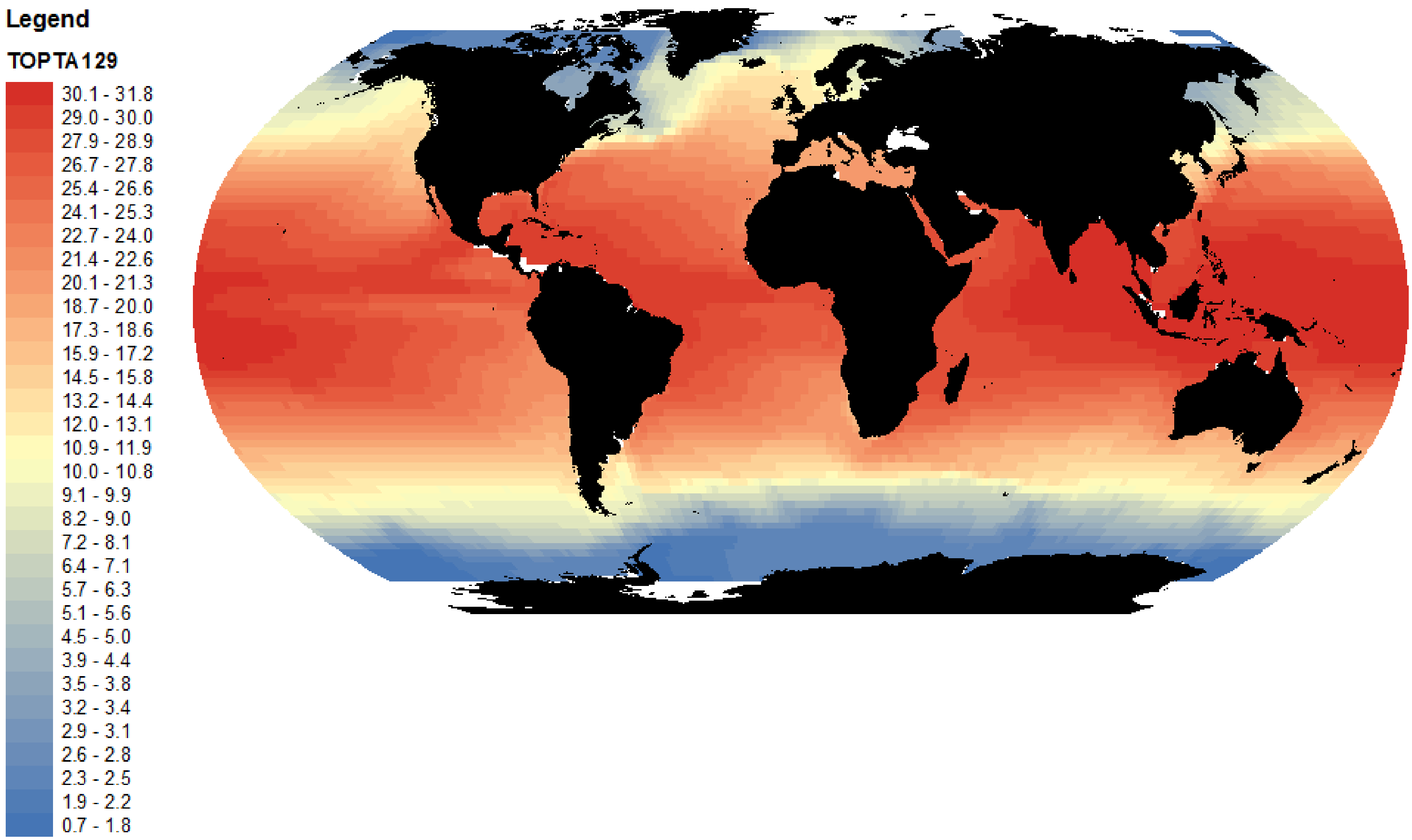

TOPTA129

[IAM, 10.0.gt.a, 9/4/2015, Run A2, Cip]

Optimum temperature, without advection (T_{LOC}*(na)*, °C)
Pop. ave. grow rate = 1.29/d

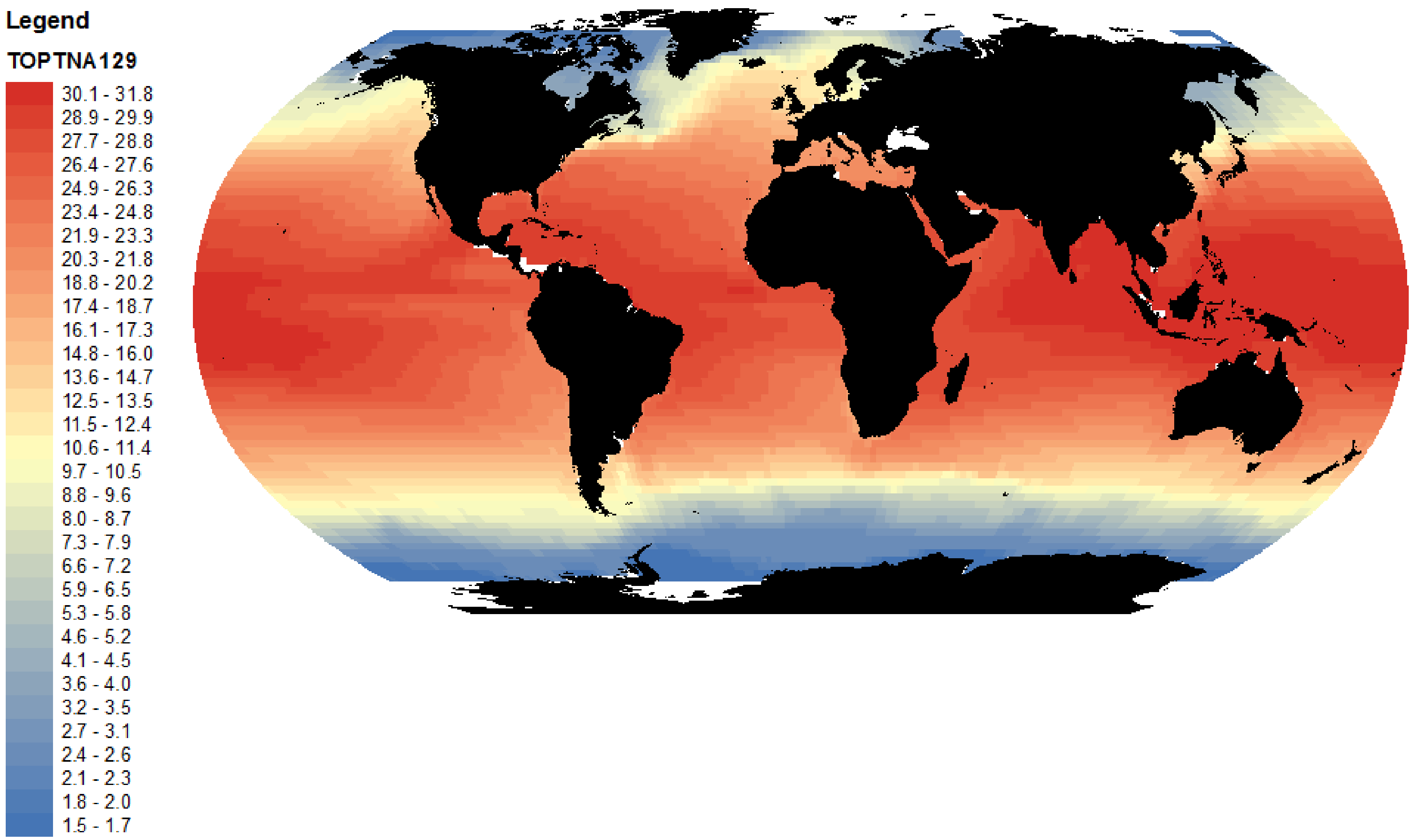

TOPTNA129

[IAM, 10.0.gt.a, 9/4/2015, Run A2, Cip]

Advective temperature differential (ΔT_{OPT}, °C)
Pop. ave. grow rate = 1.29/d

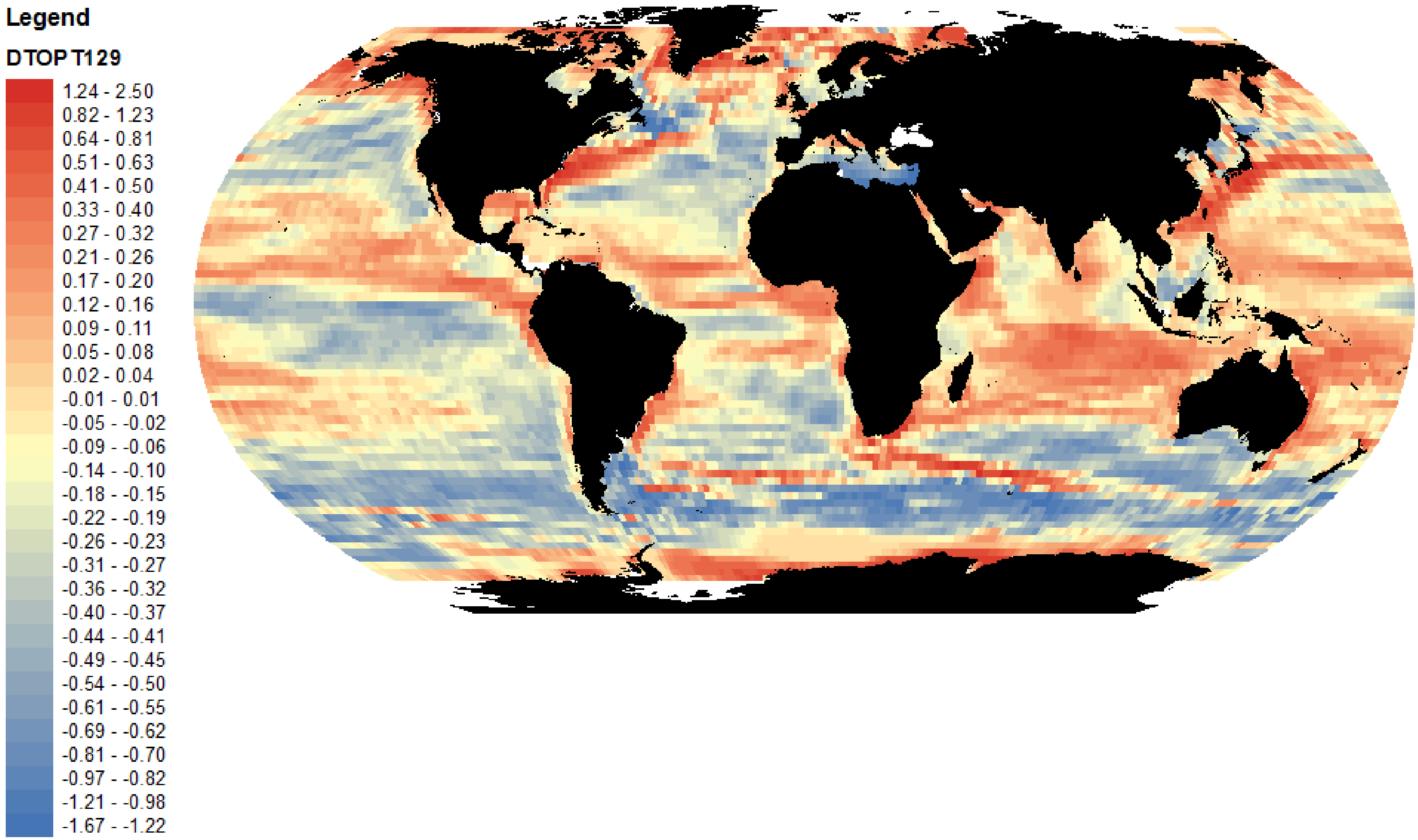

DTOPT129

[IAM, 10.0.gt.a, 9/4/2015, Run A2, Cip]
